# Long-Term Cognitive Dysfunction in Cancer Survivors

**DOI:** 10.3389/fmolb.2021.770413

**Published:** 2021-12-14

**Authors:** Zuzana Országhová, Michal Mego, Michal Chovanec

**Affiliations:** 2nd Department of Oncology, Faculty of Medicine, Comenius University and National Cancer Institute, Bratislava, Slovakia

**Keywords:** cancer-related cognitive impairment, cancer survivors, cancer treatment, risk factors, pathogenesis, biomarkers

## Abstract

Cancer-related cognitive impairment (CRCI) is a frequent side effect experienced by an increasing number of cancer survivors with a significant impact on their quality of life. Different definitions and means of evaluation have been used in available literature; hence the exact incidence of CRCI remains unknown. CRCI can be described as cognitive symptoms reported by cancer patients in self-reported questionnaires or as cognitive changes evaluated by formal neuropsychological tests. Nevertheless, association between cognitive symptoms and objectively assessed cognitive changes is relatively weak or absent. Studies have focused especially on breast cancer patients, but CRCI has been reported in multiple types of cancer, including colorectal, lung, ovarian, prostate, testicular cancer and hematological malignancies. While CRCI has been associated with various treatment modalities, including radiotherapy, chemotherapy, hormone therapy and novel systemic therapies, it has been also detected prior to cancer treatment. Therefore, the effects of cancer itself with or without the psychological distress may be involved in the pathogenesis of CRCI as a result of altered coping mechanisms after cancer diagnosis. The development of CRCI is probably multifactorial and the exact mechanisms are currently not completely understood. Possible risk factors include administered treatment, genetic predisposition, age and psychological factors such as anxiety, depression or fatigue. Multiple mechanisms are suggested to be responsible for CRCI, including direct neurotoxic injury of systemic treatment and radiation while other indirect contributing mechanisms are hypothesized. Chronic neuroinflammation mediated by active innate immune system, DNA-damage or endothelial dysfunction is hypothesized to be a central mechanism of CRCI pathogenesis. There is increasing evidence of potential plasma (e.g., damage associated molecular patterns, inflammatory components, circulating microRNAs, exosomes, short-chain fatty acids, and others), cerebrospinal fluid and radiological biomarkers of cognitive dysfunction in cancer patients. Discovery of biomarkers of cognitive impairment is crucial for early identification of cancer patients at increased risk for the development of CRCI or development of treatment strategies to lower the burden of CRCI on long-term quality of life. This review summarizes current literature on CRCI with a focus on long-term effects of different cancer treatments, possible risk factors, mechanisms and promising biomarkers.

## Introduction

Considerable advances in clinical oncology over the past decades have resulted in significant improvement of long-term survival in cancer patients. Achieving the cure in cancer means that patients become survivors who may suffer from different types of late toxicities (Miller et al., 2019). Both patients and survivors often experience changes in cognition, also called “cancer-related cognitive impairment” (CRCI), as a side effect of cancer and cancer treatment (Joly et al., 2015).

Cancer-related cognitive impairment (CRCI) is a term used to describe the decline in patients cognitive functions, such as perception, attention, language, thinking, learning and memory, action planning, understanding, reasoning and problem solving (Horowitz et al., 2018). The exact incidence and prevalence of CRCI is still unknown due to its various definitions in literature and different means of evaluation in studies. Therefore, the prevalence estimates vary widely from 15 to 75% (Wefel et al., 2004; Vardy and Tannock, 2007; Janelsins et al., 2011; Schmidt et al., 2016; Lange et al., 2019b).

Cognitive changes in cancer patients may be induced by cancer treatment or by the presence of cancer itself. Moreover, several factors may contribute to the development of CRCI, such as age, genetic predisposition, psychological and sociodemographic factors (Ahles and Root, 2018). Cognitive impairment has been described in a variety of cancer types. Treatment modalities inducing cognitive impairment include surgery, radiotherapy, chemotherapy, hormonal therapy, targeted therapy and immunotherapy (Joly et al., 2015). Acute and long-term effects on cognitive functioning resulting from cancer treatment may negatively affect the quality of life (QoL) and the ability to function in different aspects of life (Mehnert et al., 2007; Von Ah et al., 2009; Reid-Arndt et al., 2010; Hsu et al., 2013).

Cognitive dysfunction can be present in various cancers at the time of diagnosis, during cancer treatment and weeks to years after its completion. Most of the studies on cognitive changes in non-CNS cancers investigate women with breast cancer treated with chemotherapy, but recently patients with other malignancies have also been intensively studied. Cognitive impairment has been reported in patients with colorectal cancer (Cruzado et al., 2014; Vardy et al., 2015), lung cancer (Simo et al., 2015; Bromis et al., 2017), testicular cancer (Schagen et al., 2008; Wefel et al., 2014; Stouten-Kemperman et al., 2015; Amidi et al., 2017b; Chovanec et al., 2018), prostate cancer (McGinty et al., 2014; Gonzalez et al., 2015; Holtfrerich et al., 2020), ovarian cancer and other gynecological malignancies (Hess et al., 2015; Correa et al., 2017; De Rosa et al., 2021), as well as hematologic malignancies, especially after hematopoietic stem cell transplantation (HSCT) (Scherwath et al., 2013; Sharafeldin et al., 2018).

The aim of this paper is to review available literature on CRCI in non-CNS cancers, with a focus on long-term effects of different cancer treatments, possible risk factors and mechanisms of CRCI and promising biomarkers for cognitive dysfunction in cancer survivors.

## Detection and Evaluation of Cancer-Related Cognitive Impairment

Cognitive changes found in cancer survivors vary in affected domains and may be subtle, therefore, detection and evaluation of CRCI can be rather challenging (Horowitz et al., 2018). An optimal cognitive screening instrument with high sensitivity and reliability has not been yet established. The complexity of cognitive assessment in cancer survivors is specific due to two main aspects of CRCI: cognitive symptoms reported by cancer survivors (subjective) and cognitive changes evaluated by formal neuropsychological tests (objective) (Vardy et al., 2017).

Patient-reported cognitive symptoms are usually assessed with multi-item questionnaires, such as FACT-Cog (The Functional Assessment of Cancer Therapy-Cognitive Function) developed specifically to assess cognitive difficulties in cancer patients (Wagner et al., 2009; FACT-Cog, 2016). FACT-Cog questionnaire is a validated tool for self-reported measure of cognitive functions in four domains: perceived cognitive impairment (CogPCI), perceived cognitive abilities (CogPCA), quality of life affected by cognitive impairment (CogQoL) and cognitive impairment perceived by others (CogOth). Total cognitive function score is the sum of the four mentioned domains. EORTC QLQ-C30 (European Organization for the Research and Treatment of Cancer Quality of Life Questionnaire) is an integrated system for assessing the health-related QoL of cancer patients. This questionnaire is composed of five functional scales (including physical, role, emotional, cognitive and social functioning), three symptom scales, six single items and a global health status/QoL scale (Aaronson et al., 1993; EORTC QLQ, 2001).

Formal neuropsychological tests have been established as standard methods for detection of CRCI, because they provide objective assessments of various domains of cognition. The International Cognition and Cancer Task Force (ICCTF) recommends to evaluate learning, memory, processing speed and executive functions (Wefel et al., 2011; Joly et al., 2015). ICCTF suggests the use of validated neuropsychological tests, namely Hopkins Verbal Learning Test-Revised (HVLT-R) (Benedict et al., 1998), Controlled Oral Word Association Test (COWAT) of the Multilingual Aphasia Examination (Benton et al., 1994) and Trail Making Test (TMT) (Reitan, 1992). Investigators are then encouraged to supplement additional executive function tests, based on their own preferences. Cognitive impairment can be defined relative to population norms, to a control group or to an individual pretreatment functioning (a drop from baseline performance) (Horowitz et al., 2018). ICCTF recommends the following criteria for determining cognitive impairment: ≥ 2 test scores at or below −1.5 standard deviations (SDs) from the normative mean (or the mean score of an appropriate control group) or 1 test score at or below −2.0 SDs from the mean, or both (Wefel et al., 2011).

However, there is relatively weak or absent association between self-reported cognitive changes and objectively assessed cognitive performance. Many cancer survivors describe cognitive problems, but they have scores within the normal range in neuropsychological tests (Hutchinson et al., 2012; Bray et al., 2018). On the contrary, cognitive complaints are more strongly associated with other patient-reported symptoms, including anxiety, depression, fatigue or insomnia (Pullens et al., 2013; Vardy et al., 2015; Dhillon et al., 2018; Ng et al., 2018). Therefore, the assessment of these symptoms appears to be even more important.

Bray et al. (2018) suggested several factors that may be attributed to the lack of association between self-reported and objective cognitive changes in their systematic review:

- Patient’s cognitive performance was above the normal range prior to cancer diagnosis or treatment and while cognition may have declined, it subsequently remained within the normal range;
- Conditions in which neuropsychological tests are performed (structured and distraction-free environment) may not be representative of daily situations, where patients usually experience cognitive problems;
- Traditional neuropsychological tests are not sensitive enough to detect the subtle cognitive changes in CRCI;
- Self-reported and objective measures of cognitive functions may evaluate unrelated different constructs and perceived cognitive difficulties may reflect psychological distress rather than actual cognitive impairment.

Despite the differences, both subjective and objective measures for cognitive impairment are very important in clinical practice and cancer research. Both provide an essential information about the functioning of cancer patients and survivors, as well as about the impact on their quality of life. Therefore, detection and evaluation of these two aspects of CRCI should be routinely performed in cancer patients where possible (Hutchinson et al., 2012).

## Potential Risk Factors and Mechanisms of Cancer-Related Cognitive Impairment

**FIGURE 1:**
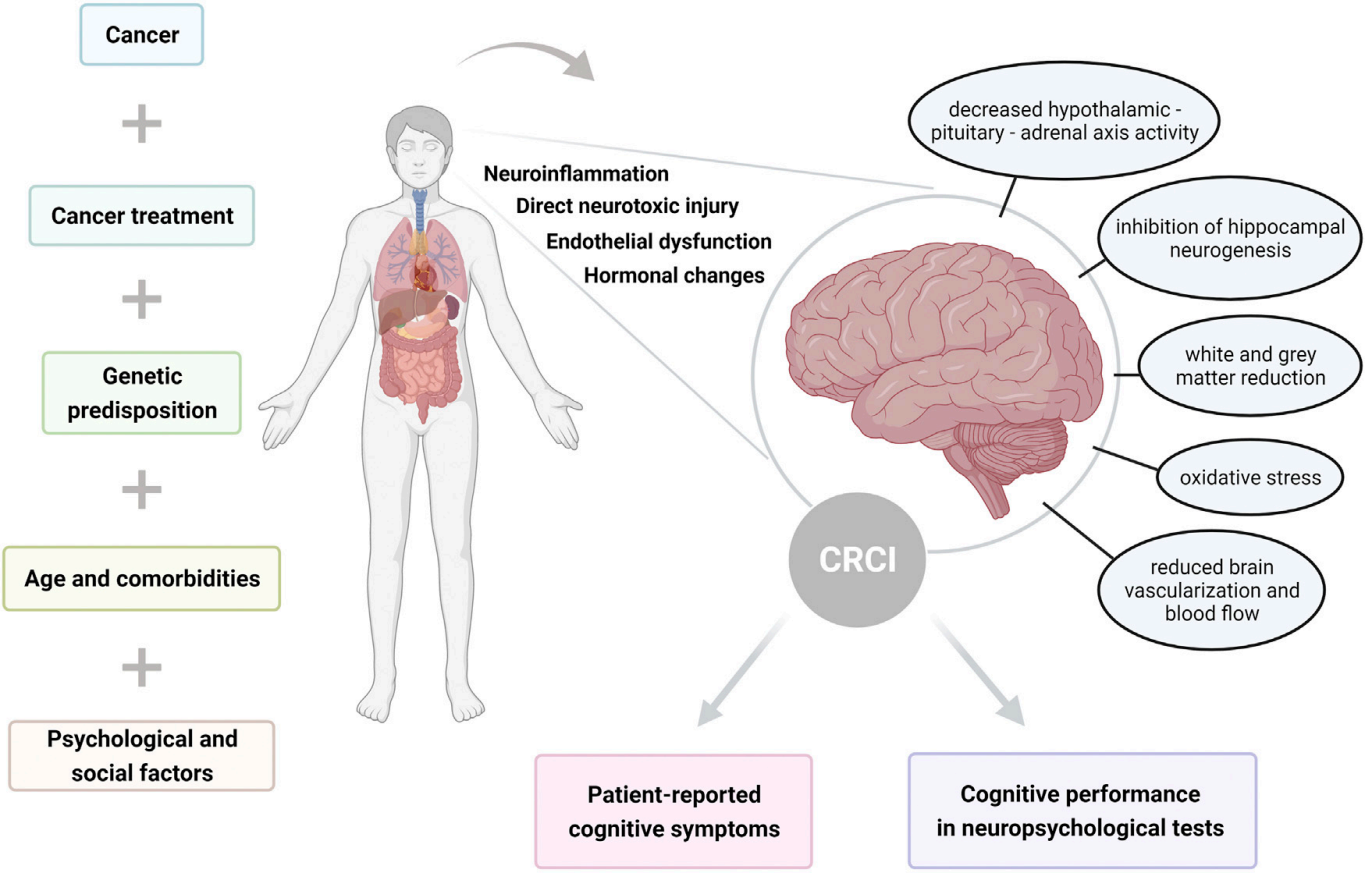
Illustration summarizing risk factors and mechanisms of cancer-related cognitive impairment (CRCI), resulting in self-reported and objectively assessed cognitive dysfunction.

The development of CRCI is probably multifactorial, although the mechanisms are still not completely understood. Therefore, it remains an area of active research. According to the studies, multiple factors can increase risk for CRCI, including genetic predisposition, age, psychological, social and demographic factors. The potential risk factors and mechanisms of CRCI are summarized in Figure 1.

### Genetic Predisposition

Known genetic factors that may predispose a patient for developing the CRCI include genes encoding apolipoprotein E (APOE) (Ahles et al., 2003; Ahles et al., 2014; Amidi et al., 2017a), catechol-O-methyltransferase (COMT) (Small et al., 2011; Cheng et al., 2016) and brain-derived neurotrophic factor (BDNF) (Ng et al., 2016). However, more are under investigation. *APOE* gene is polymorphic, with three major alleles (APOE *e2, e3, e4*). The presence of the APOE *e4* allele has been associated with increased risk for the development of Alzheimer’s disease and with traumatic brain injuries as well. Administration of chemotherapy may be considered as a type of insult to the brain, therefore *APOE e4* carriers would be more vulnerable to develop CRCI (Ahles et al., 2003). *COMT* gene determines levels of dopamine in the prefrontal cortex. *Val158Met* single-nucleotide polymorphism (SNP) represents a substitution of valine with methionine at codon 158. The presence of the Val allele is related to higher enzymatic activity compared to Met/Met homozygotes, leading to greater degradation of dopamine and its lower availability at synaptic receptors. For that reason, Val carriers may be predisposed to the development of cognitive dysfunction following cancer treatment (Small et al., 2011). *BDNF* gene encodes a neurotrophin with multiple functions in the brain. It has several known SNPs, including the most investigated *Val66Met* polymorphism (an amino acid change from valine to methionine at codon 66). Association between this SNP and neurodegenerative and mental disorders has been intensively studied, as well as its possible connection to chemotherapy-induced cognitive dysfunction (Ng et al., 2016).

Genetic risk factors and their association with CRCI are detailed in the chapter “Promising novel biomarkers of CRCI.”

### Age

Age is a well-known risk factor for many cognitive disorders and may also play a role in the development of CRCI. In addition, cognitive reserve, defined as innate and developed cognitive capacity (influenced by education, occupation and lifestyle), has also been associated with potential vulnerability to cognitive decline after brain insults. Ahles et al. (2010) studied the impact of age and cognitive reserve on cognitive functioning in breast cancer patients (*N* = 60; mean age = 51.7 years) treated with adjuvant chemotherapy, showing that older patients with lower baseline cognitive reserve performed worse in post-treatment processing speed compared with patients not exposed to chemotherapy (*p* < 0.003) and healthy controls (*p* < 0.001). Schilder et al. (2010) suggested possible age-dependency of the effects of tamoxifen on cognition, because in their study more cognitive domains were affected in women older than age 65 versus those younger than 65. Many other studies supported the association between higher age and increased risk of the development of CRCI (Mandelblatt et al., 2014; Anstey et al., 2015; Lange et al., 2016; Morin and Midlarsky, 2018; Lange et al., 2019a).

### Psychological and Other Factors

Psychological changes in cancer patients as a reaction to cancer diagnosis and its treatment are very frequent. Therefore, their potential impact on cognition has been intensively studied. Several studies found association between cognitive impairment and anxiety (Schilder et al., 2012; Pullens et al., 2013; Vardy et al., 2015; Janelsins et al., 2017; Dhillon et al., 2018), depression (Schilder et al., 2012; Ganz et al., 2013b; Danhauer et al., 2013; Pullens et al., 2013; Seliktar et al., 2015; Vardy et al., 2015; Dhillon et al., 2018), post-traumatic stress disorder (Andreotti et al., 2015; Hermelink et al., 2015) and sleeping difficulties (fatigue and insomnia) (Schilder et al., 2012; Vardy et al., 2015; Ng et al., 2018). Moreover, informing patients about cognitive side effects of the treatment may induce an increase in self-reported cognitive impairment and decrease in neuropsychological test performance among vulnerable individuals (Schagen et al., 2012; Jacobs et al., 2017). In a large study including 1,393 breast cancer survivors, 47% of them (*N* = 657) reported cognitive complaints through online FACT-Cog questionnaires. These complaints were strongly associated with chemotherapy (OR = 2.26; 95% CI = 1.67–3.05; *p* < 0.001), as well as with frequency of psychotropic treatments (OR = 1.70, 95% CI = 1.23–2.36; *p* < 0.001), post-traumatic stress symptoms (OR = 2.05, 95% CI = 1.57–2.69; *p* < 0.001) and sleep difficulties (OR = 2.41, 95% CI = 1.47–3.95; *p* < 0.001) (Boscher et al., 2020).

Patient comorbidities may also play a role in the development of cognitive dysfunction in cancer patients. Mandelblatt et al. (2014) found that comorbidity (primarily cardiovascular disease and diabetes) was strongly associated with pretreatment cognitive impairment in nonmetastatic breast cancer patients (*N* = 164; OR = 8.77; 95% CI, 2.06 to 37.4; *p* = 0.003), but not among healthy controls (*N* = 182, *p* = 0.97). Potential explanation for this can be that the comorbidity itself is associated with cognitive impairment or it increases the risk for both cancer and CRCI through chronic inflammation or acceleration of aging.

Additional factors that can influence CRCI include race, ethnicity, socioeconomic status and education. Genetic variability and differences in brain structure and function may cause diverse vulnerability to cognitive decline across racial/ethnic groups. Borenstein et al. studied frequency of *APOE e4* allele in African-American (*N* = 253) and Caucasian (*N* = 466) populations and the association with performance in five cognitive measures. *APOE e4* allele frequency in the African-American sample was 29.5% compared to 12.1% in the Caucasian sample. In Caucasians, *APOE e4* carriers performed more poorly on three of the five cognitive tests. However, in the African Americans, no association was found between the presence of the *APOE e4* allele and any cognitive outcome (Borenstein et al., 2006). A meta-analysis showed *APOE e4* genotype prevalence varies among the population diagnosed with Alzheimer’s disease (AD) by geographic region, with the highest estimates in Northern Europe and the lowest estimates in Asia and Southern Europe (Ward et al., 2012). Furthermore, Zahodne et al. found certain structural MRI predictors of cognition differed across racial/ethnic groups (*N* = 638; 29% non-Hispanic White, 36% African American, 35% Hispanic). Larger white matter hyperintensity volume was associated with worse language (*p* = 0.003) and speed/executive functioning (*p* = 0.006) among African Americans, but not among non-Hispanic Whites (both *p* > 0.1). Larger hippocampal volume was more strongly associated with better memory among non-Hispanic Whites compared to Hispanics (*p* < 0.001 vs. *p* = 0.061) (Zahodne et al., 2015). Finally, education level of patients may have impact on their cognitive reserve, as well as socioeconomic status, affecting their attitude to cancer treatment and psychological changes associated with cognitive impairment. A premorbid intellectual functioning (IQ) showed to be statistically significant predictor of cognitive impairment in a group of testicular cancer survivors (*N* = 72), with higher IQ levels being associated with lower odds of developing cognitive impairment (OR = 0.87; 95% CI: 0.81–0.95; *p* < 0.01) (Amidi et al., 2015b). Similar results were found in a group of lymphoma patients (Wouters et al., 2016).

### Mechanisms of Cancer-Related Cognitive Impairment

Based mostly on animal models, suggested mechanisms for the development of CRCI include inhibition of hippocampal neurogenesis, white matter damage, oxidative damage, immune and inflammatory processes, decreased hypothalamic-pituitary-adrenal axis activity, reduced brain vascularization and blood flow (Seigers and Fardell, 2011; Seigers et al., 2013). Other possible mechanisms are direct neurotoxicity with damage of brain neuronal cells from cytostatic agents that can cross the blood-brain barrier or hormonal changes induced by chemotherapy, leading to cognitive impairment (Ahles and Saykin, 2007; Merriman et al., 2013). Probably one of the dominant processes in cognitive impairment is chronic inflammation and neuroinflammatory pathways. Chemotherapy that disrupts cellular processes and cell division can lead to increased levels of inflammatory components during and after treatment administration, especially pro-inflammatory cytokines (e.g., IL-1, IL-6, and TNF-alpha) and cytokine receptors (e.g., sTNFRI and sTNFRII). Several studies have demonstrated an association between markers of inflammation and decreased cognitive function in cancer patients (Kesler et al., 2013b; Cheung et al., 2015; Lyon et al., 2016; Williams et al., 2018). A few pre-clinical studies evaluated changes in the expression of inflammation-related genes associated with the development of CRCI (Kovalchuk et al., 2016; Allen et al., 2019; Bagnall-Moreau et al., 2019). Oppegaard et al. in their recent clinical study evaluated differentially expressed genes and perturbed inflammatory pathways across two independent samples of cancer patients who did and did not report cognitive difficulties. The 16-item Attentional Function Index (AFI) was used for the assessment of self-reported CRCI. Approximately half of the patients in each sample had AFI scores indicating low levels of cognitive function. A gene expression of total RNA isolated from peripheral blood of the 717 patients was quantified for 357 patients (Sample 1) using RNA-sequencing and for 360 patients (Sample 2) using microarray. Twelve signaling pathways were significantly perturbed between the patients with low and high AFI scores, five of which were signaling pathways related to inflammatory mechanisms: cytokine-cytokine receptor interaction, mTOR (mechanistic target of rapamycin), MAPK (mitogen-activated protein kinase), IL-17 (interleukin-17) and TNF (tumor necrosis factor) signaling pathways (all *p* < 0.05). This study was the first to describe abnormalities in inflammatory pathways associated with CRCI, supporting an important role of inflammation in its pathogenesis (Oppegaard et al., 2021).

**FIGURE 2:**
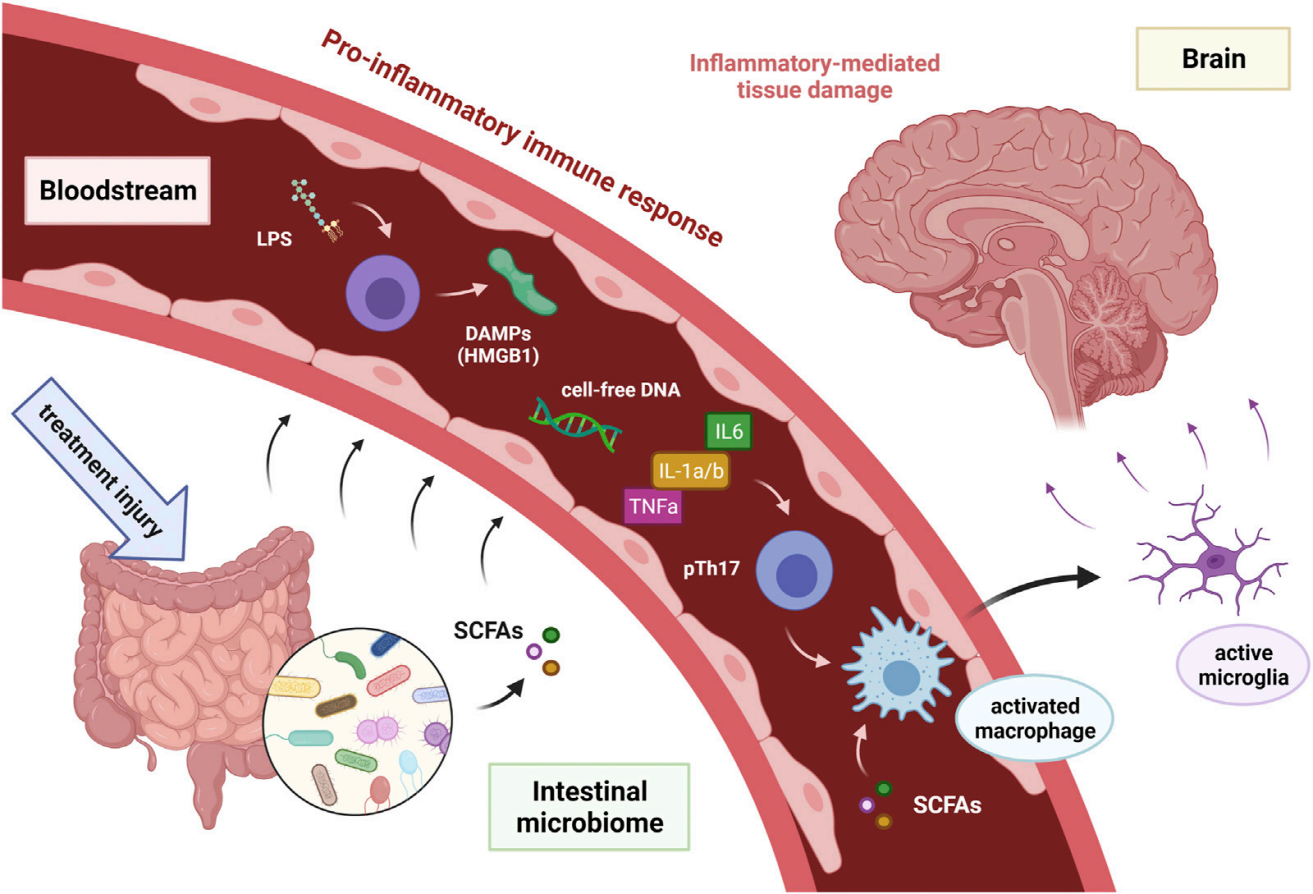
Hypothetical model of proposed immune-related mechanism of cancer treatment induced cognitive impairment in cancer survivors [adopted from Ciernikova et al. (2021)]. Abbreviations DAMPs = damage-associated molecular patterns; HMGB 1 = high-mobility group box 1; IL-1a/b = interleukin 1a and 1b; IL6 = interleukin 6; LPS = intestinal microbiota associated lipopolysaccharide; SCFAs = short-chain fatty acids produced by intestinal microbiota; TNFa = tumor necrosis factor-alpha.

In recent years, a role of gut microbiome modulating neurochemical pathways and brain functions through the interconnected gut-brain axis has been the subject of intensive research. Although the exact mechanisms involved in the communication between the gut microbiome and the brain are still not known, there are several possible pathways through which the intestine can influence brain function (Borre et al., 2014). Differences in the gut microbiome have been observed in Alzheimer’s disease, Parkinson’s disease and autism spectrum disorders (Hasegawa et al., 2015; Zhang et al., 2017; Sharon et al., 2019). Therefore, it may also play an important role in the development of CRCI in patients receiving chemotherapy or radiotherapy. However, cognitive impairment in neurodegenerative diseases and in cancer may have separate pathogenetic mechanisms. We have previously proposed a dysregulation of the microbiota-gut–brain communication pathways as a possible immune-related mechanism of CRCI **(** Figure 2 **)** (Ciernikova et al., 2021). Cancer treatment causes cytotoxic or radiation damage to the physiological gut microbiome, leading to intestinal dysbiosis and increased release of lipopolysaccharides (LPS) from the cell wall of Gram-negative bacteria. Subsequent intestinal barrier disruption results in translocation of whole bacteria or bacterial LPS, damage-associated molecular patterns (DAMPs) and microbiota-derived metabolites (e.g., SCFA), as well as cell-free DNA into the bloodstream. This leads to the proinflammatory immune response and activation of microglia in the hippocampus, resulting in neuroinflammation, oxidative stress and neuron loss associated with cognitive impairment (Figure 2) (Chen et al., 2008; Catorce and Gevorkian, 2016; Ciernikova et al., 2021).

## The Impact of Cancer Treatment on Cognitive Functioning

### Cancer-Related Cognitive Impairment After Surgery and Prior to Systemic Treatment

Various studies in patients with non-CNS cancers have shown the presence of cognitive dysfunction after surgery, before the administration of systemic treatment or even before any cancer treatment. The first prospective longitudinal study evaluating cognition in breast cancer patients was performed by Wefel et al. (2004). This study included 18 women with non-metastatic breast cancer (mean age = 45.4 ± 6.7) after surgery, who were evaluated with neuropsychological tests and self-reporting questionnaires before the start of adjuvant chemotherapy (baseline), short-term and long-term post-chemotherapy (approximately 6 and 18 months after baseline). Thirty-three percent of women exhibited a cognitive impairment prior to receiving chemotherapy, especially in verbal learning and memory. Sixty-one percent of patients exhibited cognitive decline short-term post-chemotherapy compared to baseline (most affected domains were attention, learning, and processing speed). Half of the patients who experienced decline in cognitive functions demonstrated an improvement in the long-term post-chemotherapy, whereas half of them remained stable. Ahles et al. found breast cancer patients with stage I-III disease (*N* = 110; age = 54.1 ± 8.1 years) had a significantly lower than expected overall cognitive performance (*p* = 0.002) in neuropsychological tests (22%) following surgery but prior to adjuvant treatment, as compared to patients with noninvasive breast cancer (stage 0) (0%) and healthy controls (4%) (Ahles et al., 2008). A case-control study in elderly patients with breast cancer showed similar neuropsychological domain scores in patient cases and controls before the start of adjuvant treatment. However, among patient cases (*N* = 164), women with stage II-III disease had lower executive function scores compared to those with stage 0-I disease (*p* = 0.05) (Mandelblatt et al., 2014). Lange et al. assessed cognitive functions in elderly patients with early-stage breast cancer (N = 123; age = 70 ± 4 years) before adjuvant treatment and reported objective cognitive dysfunction in 41% of patients compared to healthy population norms (*p* < 0.0001). Verbal episodic memory was impaired in the highest proportion of patients (21%) (Lange et al., 2014).

Menning et al. (2015) combined neuropsychological testing, patient-reported outcomes and multimodal MRI to examine pretreatment cognition, brain functions and structure in breast cancer patient after surgery. This study showed overall cognitive performance was lower in the pre-chemotherapy groups (one about to receive chemotherapy and one not indicated to undergo chemotherapy), compared to healthy controls (*p* = 0.021). In addition, patients demonstrated prefrontal hyperactivation with increasing task difficulty on a planning task compared to healthy controls. However, the cognitive and imaging data showed that symptoms of fatigue were associated with the observed abnormalities and the differences between groups were no longer significant when fatigue was accounted for (Menning et al., 2015). Many other neuropsychological (Hermelink et al., 2007; Quesnel et al., 2009; Wefel et al., 2010; Jansen et al., 2011; Yao et al., 2016) and imaging (Cimprich et al., 2010; Scherling et al., 2011; McDonald et al., 2012; Scherling et al., 2012; Lopez Zunini et al., 2013) studies in breast cancer patients showed similar results, pointing to the existence of pretreatment cognitive and brain dysfunction, although the exact mechanism is still under investigation.

The presence of cognitive impairment prior to systemic therapy has been shown in colorectal cancer as well. Cruzado et al. (2014) showed 37% (30/81) of patients with colorectal cancer had cognitive impairment in the pre-chemotherapy evaluation, mainly in processing speed and psychomotor executive functions. Vardy et al. (2014) found that 45% (126/281) of patients with early-stage colorectal cancer and 47% (31/66) of patients with metastatic disease had cognitive impairment before or after surgery compared to 15% (11/72) of healthy controls (OR = 4.51, 95% CI 2.28–8.93; *p* < 0.001 and OR 4.91, 95% CI 2.20–10.97; *p* < 0.001, respectively). Attention/working memory, verbal learning/memory and complex processing speed were the most affected cognitive domains. In addition, women with early-stage colorectal cancer had greater cognitive impairment than men [55/105 (52%) versus 71/176 (40%), *p* < 0.050].

A recent study assessed presence of cognitive impairment in newly orchiectomized testicular cancer patients and explored the structural brain networks, endocrine status and selected genotypes. Patients (*N* = 40) performed poorer on 6 out of 15 neuropsychological tests compared to healthy controls (*N* = 22). The proportion of cognitive impairment was also higher in patients’ group (65 vs. 36%; *p* = 0.04). Global brain network analysis revealed no differences between these two groups, but regional analysis indicated differences in node degree and betweenness centrality in several regions (*p* < 0.05), that was inconsistently associated with cognitive performance. In addition, no associations were found for APOE, BDNF or testosterone levels (Buskbjerg et al., 2021).

### Chemotherapy and Cognitive Impairment

Multiple trials studied effects of chemotherapy (adjuvant treatment or systemic treatment for metastatic disease) on cognitive functions. Studies have been focused on patients with breast, ovarian, testicular, colorectal cancer and others (Schagen et al., 2008; Cruzado et al., 2014; Wefel et al., 2014; Hess et al., 2015; Joly et al., 2015; Stouten-Kemperman et al., 2015; Vardy et al., 2015; Amidi et al., 2017b; Correa et al., 2017; Chovanec et al., 2018). Cognitive changes were usually evaluated before the initiation of chemotherapy, shortly after its completion and in different subsequent intervals. Many studies showed a self-reported or objectively evaluated cognitive decline after chemotherapy.

A prospective longitudinal study assessed a cognitive function in 581 early breast cancer patients (stage I–IIIC) treated with adjuvant chemotherapy. Cognitive function was assessed using FACT-Cog questionnaires at prechemotherapy, postchemotherapy and at a 6-months follow-up and compared to 364 healthy controls. Patients reported significantly greater cognitive difficulties from prechemotherapy to postchemotherapy compared with controls as well as from prechemotherapy to 6-months follow-up (all *p* < 0.001) (Janelsins et al., 2017).

On the contrary, a long-term follow-up study (7–9 years after primary surgery) with almost 1,900 breast cancer survivors found no difference in subjective cognitive impairment between women who had received adjuvant systemic therapies and those who had not (Amidi et al., 2015a). Cognitive impairment was assessed with the Cognitive Failures Questionnaire only at 7–9 years follow-up, therefore it could not explore cognitive changes over further time points. In addition, an initial cognitive level before cancer treatment was unknown. However, there are also other studies that did not show negative impact of neoadjuvant (Hermelink et al., 2007) and adjuvant (Debess et al., 2010; Tager et al., 2010) chemotherapy on cognitive functions in women with nonmetastatic breast cancer.

Hess et al. (2015) evaluated cognitive changes in ovarian cancer patients (*N* = 231) with patient-reported questionnaires prior to chemotherapy, prior to cycle four, after cycle six, and 6 months after completion of primary therapy. Twenty-five percent (55/218) of patients exhibited cognitive impairment in at least one domain at the cycle 4 time point. After cycle 6 and at 6-months follow up time points, 21.1% (44/208) and 17.8% (30/169) of patients, respectively, demonstrated impairment in at least one cognitive domain. However, there were statistically significant improvements in processing speed (*p* < 0.001) and attention (*p* < 0.001) from baseline through the 6-months follow up time period.

Several studies in patients with testicular germ cell tumors (GCT) showed possible negative effect of cisplatin-based chemotherapy on their cognition (Schagen et al., 2008; Amidi et al., 2015b; Stouten-Kemperman et al., 2015; Chovanec et al., 2018), while other studies did not (Pedersen et al.,2009; Skaali et al., 2011; Whitford et al., 2020). Schagen et al. (2008) evaluated cognitive complaints of GCT survivors with median follow-up 3 years. Seventy patients were treated with BEP (bleomycin + etoposide + cisplatin) chemotherapy after orchiectomy, 57 patients were treated with radiotherapy after orchiectomy and 55 patients were treated with orchiectomy only. The study showed a cognitive impairment in patients treated with orchiectomy + chemotherapy versus orchiectomy alone (*p* = 0.038). However, there were no significant changes between groups treated with orchiectomy + chemotherapy versus orchiectomy + radiotherapy (*p* = 0.7) and orchiectomy + radiotherapy versus orchiectomy alone (*p* = 0.07). A study by Amidi et al. (2015b) evaluated 72 GCT survivors 2–7 years post-treatment with neuropsychological tests. GCT survivors scored significantly lower than norms (*p* < 0.01) on a majority of neuropsychological subtests (9/12), with 62.5% of survivors (45/72) categorized as having cognitive impairment. Our prospective study (Chovanec et al., 2018) evaluated a long-term cognitive functioning in GCT survivors using FACT-Cog questionnaires after median 10 years of follow-up. One hundred and fifty-five survivors were treated with orchiectomy and subsequent cisplatin-based chemotherapy, radiotherapy or both, compared to survivors treated with orchiectomy only. Any treatment beyond orchiectomy resulted in significantly greater cognitive difficulties on the overall cognitive function score. Radiotherapy was associated with cognitive declines in overall cognitive functioning and in subscales for perceived cognitive impairment and cognitive impairment perceived by others (both *p* < 0.05). Chemotherapy + radiotherapy or radiotherapy groups showed an impairment in all cognitive functioning domains in comparison with controls (all *p* < 0.05).

Wouters et al. (2016) studied cognitive changes in patients with non-Hodgkin or Hodgkin lymphoma who had been treated with standard dose or supplementary high dose chemotherapy. This study did not show worse overall cognitive functioning of patients (*N* = 106) compared to matched controls (*N* = 53). However, a subgroup of 16% of patients had a poorer neuropsychological performance, that could have been caused by lower education and lower estimated premorbid intelligence.

According to meta-analyses of studies on cognitive dysfunction in various cancer patients treated with chemotherapy, the most affected cognitive domains were attention, memory, verbal and visuospatial ability, executive functions and processing speed (Jim et al., 2012; Hodgson et al., 2013; Lindner et al., 2014). Hodgson et al. (2013) found a negative relationship between the level of cognitive impairment and the duration of treatment (*r* = - 0.63, *p* < 0.01). The level of cognitive impairment was represented by the mean effect size and duration of treatment was operationalized by the number of cycles of chemotherapy received. Furthermore, a study by Collins et al. (2013) showed the cognitive impairment worsened with cumulative chemotherapy exposure in early-stage breast cancer patients (*N* = 60) treated with adjuvant chemotherapy, supporting a dose-response relationship.

Longitudinal studies found that cognitive decline after chemotherapy can persist for months or years. Koppelmans et al. (2012a) evaluated breast cancer survivors (*N* = 196) more than 20 years after adjuvant chemotherapy and their cognitive performance was significantly worse than that of the reference group on neurocognitive tests of immediate (*p* = 0.015) and delayed verbal memory (*p* = 0.002), processing speed (*p* < 0 0.001), executive functioning (*p* = 0.013), and psychomotor speed (*p* = 0.001). However, a study by Jansen et al. (2011) showed a potential reversibility of cognitive changes induced by adjuvant chemotherapy in early-stage breast cancer patients (*N* = 71). The reversibility of cognitive impairment was not described in all cognitive domains. Cognitive functions were assessed prior to adjuvant chemotherapy, 1 week after the last cycle of chemotherapy and subsequently after 6 months. Significant cognitive decreases immediately after completing the chemotherapy were followed by improvements 6 months after chemotherapy in the cognitive domains of visuospatial skills (*p* < 0.001), attention (*p* = 0.022), delayed memory (*p* = 0.006) and motor function (*p* = 0.043), while executive function, immediate memory and language scores did not change (*p* < 0.05).

Interestingly, a large online survey including more than 1,610 cancer survivors who have finished their curative treatments for different types of cancers (median post-treatment time was 2.83 years), found that cognitive complaints were reported by 75% of participants, and three quarters of them admitted that cognitive difficulties had an impact on their work resumption (Lange et al., 2019b). In another online survey 47.2% of almost 1,400 breast cancer survivors reported cognitive complaints, particularly after chemotherapy (Boscher et al., 2020).

### Hormonal Therapy and Cognitive Impairment

A negative impact of hormonal therapy on cognition was shown in several studies on women with breast cancer (Castellon et al., 2004; Schilder et al., 2010; Ganz et al., 2014; Bender et al., 2015; Wagner et al., 2020; Yamamoto et al., 2020) as well as on men with prostate cancer (McGinty et al., 2014; Gonzalez et al., 2015). Castellon et al. (2004) compared the cognitive functioning in breast cancer survivors who received adjuvant chemotherapy (*N* = 18) or chemotherapy + tamoxifen (*N* = 18) to patients treated with surgery only (*N* = 17). A group of patients receiving only chemotherapy performed significantly worse in the domains of verbal learning (*p* = 0.03), visuospatial functioning (*p* = 0.005) and visual memory (*p* = 0.01) than patients treated with surgery only. In addition, patients who received both chemotherapy and tamoxifen showed the greatest cognitive impairment in neuropsychological tests. These findings were supported by recent study (Wagner et al., 2020) comparing patient-reported cognitive impairment among women with early breast cancer (*N* = 552) treated with chemotherapy + hormonal therapy versus hormonal therapy alone (58% received an aromatase inhibitor as initial endocrine therapy and 37% received tamoxifen). In this study, cognitive functions were assessed using the FACT-Cog questionnaire at baseline, 3, 6, 12, 24 and 36 months, showing adjuvant chemotherapy + hormonal therapy was associated with significantly greater CRCI compared to hormonal therapy alone at 3 and 6 months (*p* < 0.001 and *p* = 0.02, respectively). However, no significant differences were observed at 12 months and beyond. Schilder et al. (2010) compared the impact of adjuvant tamoxifen and exemestane on cognitive functions in postmenopausal breast cancer patients. Eighty tamoxifen users and 99 exemestane users were assessed with neuropsychological tests before the start and after 1 year of adjuvant hormonal treatment. This study showed that 1 year of adjuvant therapy with tamoxifen was associated with significantly lower scores in verbal memory (*p* < 0.01) and executive functions (*p* = 0.01) compared to the healthy controls, and lower scores on information processing speed (*p* = 0.02), compared to exemestane. However, exemestane users did not perform significantly worse than healthy controls on any cognitive domain. A longitudinal study by Bender et al. (2015) evaluated effects of anastrozole on the cognition in early-stage breast cancer patients for the first 18 months of treatment (chemotherapy + anastrozole, *N* = 114; and anastrozole alone, *N* = 173). This study found patients had poorer executive functioning than healthy controls at nearly all time points (*p* < 0.0001 to *p* = 0.09). Furthermore, women treated with anastrozole alone showed a second decline in concentration and working memory from 12 to 18 months after initiation of therapy (*p* < 0.0001 and *p* = 0.02, respectively).

Nevertheless, there are studies that did not show significant negative impact of treatment with selective estrogen receptor modulators or aromatase inhibitors on cognition (Danhauer et al., 2013; Hurria et al., 2014; Le Rhun et al., 2015; Phillips et al., 2016). A recent prospective longitudinal study by Van Dyk et al. (2019) evaluated cognitive functions of early-stage breast cancer survivors (*N* = 189) treated with hormonal therapy (tamoxifen or aromatase inhibitors) prior to initiation of therapy, after 6 months, 12 months and 3–6 years of therapy. Authors did not find any cognitive differences over the follow-up time between women taking hormonal therapy or not. However, Hurria et al. (2014) found that even though there was no significant decline in cognitive function among elderly breast cancer patients receiving aromatase inhibitors (*N* = 32) compared to healthy controls, there was an increased metabolic activity on PET scans between baseline and 6 months follow-up in medial temporal and cerebellar regions. The most significant increase was observed in the right medial temporal lobe (*p* < 0.0005).

The influence of androgen deprivation therapy (ADT) on cognitive functions in prostate cancer patients is also intensively studied. Several studies did not prove significant negative effects of ADT on cognition (Wiechno et al., 2013; Alibhai et al., 2017; Morote et al., 2017; Marzouk et al., 2018), but there are multiple studies that found an association between ADT and cognitive impairment in prostate cancer patients (Gonzalez et al., 2015; Gunlusoy et al., 2017; Holtfrerich et al., 2020; Thiery-Vuillemin et al., 2020). According to the meta-analysis of 14 studies, the most significant cognitive impairment was in visuomotor functions (McGinty et al., 2014). A large prospective study by Gonzalez et al. (Gonzalez et al., 2015) evaluated prostate cancer patients receiving ADT (*N* = 58) for 12 months and compared them to prostate cancer patients without the ADT and healthy controls, using neuropsychological tests. Men treated with ADT had lower cognitive performance within 6 and 12 months after starting the ADT in comparison with both control groups (*p* < 0.05 for both comparisons). Interestingly, there are a few retrospective studies exploring potential association between the ADT and neurodegenerative diseases (especially Alzheimer’s disease) in large electronic databases. Two large analyses by Nead et al. (2016, 2017) found positive association between the ADT and the risk of Alzheimer’s disease (*N* = 16,888; HR = 1.88; 95% CI, 1.10 to 3.20; *p* = 0.021) and between the ADT and the risk of all types of dementia (*N* = 9,272; HR = 2.17; 95% CI, 1.58–2.99; *p* < 0.001). A retrospective study among elderly prostate cancer patients (*N* = 154,089) found that the ADT exposure was associated with subsequent diagnosis of Alzheimer’s disease or dementia during the longest mean follow-up of 8.3 years (Jayadevappa et al., 2019). However, the largest population-based cohort study evaluating 1.2 million prostate cancer patients with a mean follow-up of 5.5 years did not find increased risk of Alzheimer’s disease or dementia for men receiving ADT (Baik et al., 2017). Discrepancies in these findings might have been due to the different lengths of follow-up or lack of information about other risk factors of Alzheimer’s disease and dementia.

### Targeted Therapy and Cognitive Impairment

Current research on CRCI is majorly focused on traditional chemotherapy-induced cognitive impairment, but there are several trials studying potential impact of targeted therapies on cognition. A review by Ng et al. (2014) described the importance of vascular endothelial growth factor (VEGF) for CNS and cognitive functioning through its effects on neurogenesis and neuroprotection, and possible neurotoxic effect of VEGF inhibitors on cancer patients’ cognitive functions. Mulder et al. (2014) evaluated a cognitive impairment in patients with metastatic renal cell cancer (mRCC) or gastrointestinal stromal tumor (GIST) during the treatment with VEGFR tyrosine kinase inhibitors sunitinib or sorafenib (*N* = 30), compared to patients with mRCC not receiving the systemic treatment (patient controls, *N* = 20) and healthy controls (*N* = 30). This study found that both patient groups (with or without systemic treatment) performed significantly worse in the neuropsychological tests, with executive functions (*p* = 0.005 and *p* = 0.049, respectively), learning and memory (*p* = 0.0001 and *p* = 0.019, respectively) as the most affected domains. These findings suggested possible negative effects of treatment with VEGFR tyrosine kinase inhibitors on cognition. However, the cognitive decline in patient groups might have been influenced by anxiety or depression due to treatment as well as fatigue. Another study examined cognitive effects of antiangiogenic therapies in patients with mRCC (*N* = 75) and their relation with fatigue. Cognitive changes were observed in 31% of patients, especially in working memory and information-processing speed. A relationship between fatigue and cognitive complaints was observed (*p* < 0.05), but not with objective cognitive decline (Joly et al., 2016).

A cognitive impairment has not been specifically described in patients receiving cancer immunotherapy with immune checkpoint inhibitors (CPI) or chimeric antigen receptor T-cells (CAR T-cell therapy), even though neurological immune-related adverse events associated with immunotherapy are well known. A review by Joly et al. (2020) summarized potential biological and pathophysiological effects of immunotherapy on cognitive functions in cancer patients. The immune-related neurological adverse events (nAEs) induced by CPI are uncommon, with very heterogeneous clinical spectrum (including encephalopathies, meningoradiculoneuritis, Guillain-Barré like syndromes, myasthenic syndromes and more), usually grade 1–2. An overall incidence of nAEs was described <4% after treatment with anti-CTLA-4 agents, 6% with anti-PD-1 agents and 12% with combination therapy (Cuzzubbo et al., 2017). CAR T-cell therapy causes frequent and severe neurological disorders that appear soon after the infusion. A study with animal models combining radiotherapy and immunotherapy showed behavioral alterations and cognitive impairment accompanied by an increased microglial activation and changes in proinflammatory cytokines (McGinnis et al., 2017).

The selected cancer treatments and their effect on cognitive functions with associated possible mechanisms of CRCI are summarized in Table 1.

**TABLE 1:** Selected cancer treatments and affected cognitive functions with associated possible mechanisms of CRCI.

Cancer treatment	Affected cognitive functions	Possible mechanisms
**CHEMOTHERAPY**		
Doxorubicin-based regimens	Clinical studies: executive function, language, memory (short-term verbal memory), processing speed Eide and Feng (2020);	Inflammation
		Increased oxidative stress DNA damage
		Mitochondrial dysfunction
		Activation of apoptosis
	Animal studies: short-term memory (Matsos and Johnston (2019)	Damaged neurogenesis
		Synaptic dysplasia
		Down-regulation of neurotransmitters
		Epigenetic alterations Ongnok et al. (2020); Du et al. (2021)
Cisplatin-based regimens	Clinical studies: overall cognitive decline, verbal learning, memory Amidi et al. (2017b); Chovanec et al. (2018)	Direct damage of oligodendrocytes
		Mitochondrial dysfunction; Increased oxidative stress
	Animal studies: short- and long-term memory, executive function Lomeli et al. (2017); Matsos and Johnston (2019)	Loss of microtubule stabilization—increasing phosphorylation of tau protein Ongnok et al. (2020)
		Damage of gut microbiome leading to neuroinflammation (Ciernikova et al. (2021)
Taxane-based regimens	Clinical studies: attention, concentration, executive function Ibrahim et al. (2021)	InsP3R calcium pathway and impaired neuronal morphology Chen et al. (2021); Nguyen et al. (2021)
	Animal studies: short-term spatial memory Callaghan and O'Mara (2015); Nguyen et al. (2021)	Reduction of hippocampal neurogenesis *via* down-regulation of vesicular zinc Lee et al. (2017)
Methotrexate	Clinical studies: leukoencephalopathy (childhood ALL) Bhojwani et al. (2014)	Activation of microglia and neuroinflammation
	Animal studies: spatial and visual memory, executive function Wen et al. (2018)	Decrease in the number of oligodendrocytes and in the extent of myelination—disruption of white matter
		Suppression of neurogenesis in the hippocampus Geraghty et al. (2019); Gibson et al. (2019); Berlin et al. (2020)
**HORMONAL THERAPY**		
SERM Aromatase inhibitors	Clinical studies: verbal memory, executive function, processing speed Schilder et al. (2010), working memory and concentration Bender et al. (2015)	Decline in levels of estrogen and its effects on brain, inducing endocrine disorders and disbalance in hypothalamo-pituitary-adrenal axis Merriman et al. (2013)
ADT	Clinical studies: visuomotor ability McGinty et al. (2014); executive function Gonzalez et al. (2015)	Reduced levels of testosterone and its impact on cognition Nelson et al. (2008)
		Acceleration of age-related brain changes Plata-Bello et al. (2019)
**TARGETED THERAPY**		
VEGF inhibitors	Clinical studies: executive function, learning, memory Mulder et al. (2014); Joly et al. (2016)	Impaired neurogenesis, neuroprotection, and cerebral blood flow
		Inhibition of long-term potentiation Ng et al. (2014)
Immune checkpoint inhibitors	Clinical studies: headaches, encephalopathy, meningitis, hypophysitis (immune-related nAEs) Joly et al. (2020)	Changes in proinflammatory cytokines and increased microglial activation leading to neuroinflammation McGinnis et al. (2017); Joly et al. (2020)
CAR T-cell therapy		

Abbreviations ADT = androgen deprivation therapy; ALL = acute lymphoblastic leukemia; CAR = chimeric antigen receptor; InsP3R = inositol trisphosphate receptor; nAEs = neurological adverse events; SERM = selective estrogen receptor modulator; VEGF = vascular endothelial growth factor

### Radiotherapy and Cognitive Impairment

Treatment of both primary and metastatic brain tumors includes brain irradiation (partial or whole brain radiotherapy) and its association with cognitive changes has been known for a long time. The cognitive decline after whole brain radiotherapy may manifest several months to years following radiation exposure and may progressively worsen (Monje and Dietrich, 2012). The most affected cognitive domains are attention, learning and memory, processing speed and executive function (McDuff et al., 2013). Radiation-induced cognitive impairment involves multiple mechanisms, such as damage of different neural cell types, structural and functional changes in the brain blood vessels and glial cells, reduction of neurogenesis in the hippocampus, thus altering of neuronal function and inducing neuroinflammation (Makale et al., 2017).

Radiotherapy for localized or locally advanced disease has been a mainstay of cancer treatment for several decades. However, the impact of radiation on cognitive function in early non-CNS cancer is relatively understudied. While the majority of studies in patients with localized breast cancer evaluated an impact of chemotherapy or chemoradiotherapy, only a few studies focused on the adverse cognitive effects of radiation therapy alone. A study by Quesnel et al. (2009) compared the effect of adjuvant chemotherapy and adjuvant radiotherapy on cognitive functioning in breast cancer patients (*N* = 81). Forty-one patients received chemotherapy, that was subsequently followed by radiotherapy in 38 patients. Forty patients received radiotherapy without chemotherapy. Patients were evaluated using different neuropsychological tests at baseline, after treatment and at a 3-months follow-up. This study showed that both chemotherapy and radiotherapy group performed worse in neuropsychological tests compared to healthy controls. (Shibayama et al., 2014, 2019) showed that breast cancer patients exposed to adjuvant regional radiotherapy might develop the cognitive impairment several months after the treatment. However, the cognitive decline restored approximately 3 years after the treatment. Additionally, the presence of cognitive changes correlated with changes in plasma IL-6 levels (increased at the time of the cognitive decline and decreased after restoration).

Radiotherapy to retroperitoneal lymph nodes in patients with seminomatous testicular germ cell tumors (GCT) has been widely used for stage I or II disease (Chung and Warde, 2011). While the adjuvant radiotherapy is no more the recommended therapeutic approach, the irradiation of retroperitoneal lymph nodes up to 3 cm in size is still accepted for stage II disease (Gilligan et al., 2019). Our prospective study (Chovanec et al., 2018) evaluated a long-term cognitive functioning in GCT survivors (with a median follow-up period 10 years) treated with cisplatin-based chemotherapy, radiotherapy or both, in comparison to survivors treated with orchiectomy only, using FACT-Cog questionnaires. Radiotherapy was associated with declines in overall cognitive function score and in two cognitive domains. Survivors treated with radiotherapy and radiotherapy + chemotherapy reported the statistically significant decline in all cognitive domains compared to patients treated with orchiectomy only (all *p* < 0.05), as well as in overall cognitive function score (mean score ±SEM; 106.3 ± 4.1 vs. 119.8 ± 2.7; *p* = 0,01).

## Promising Novel Biomarkers of Cancer-Related Cognitive Impairment

**FIGURE 3:**
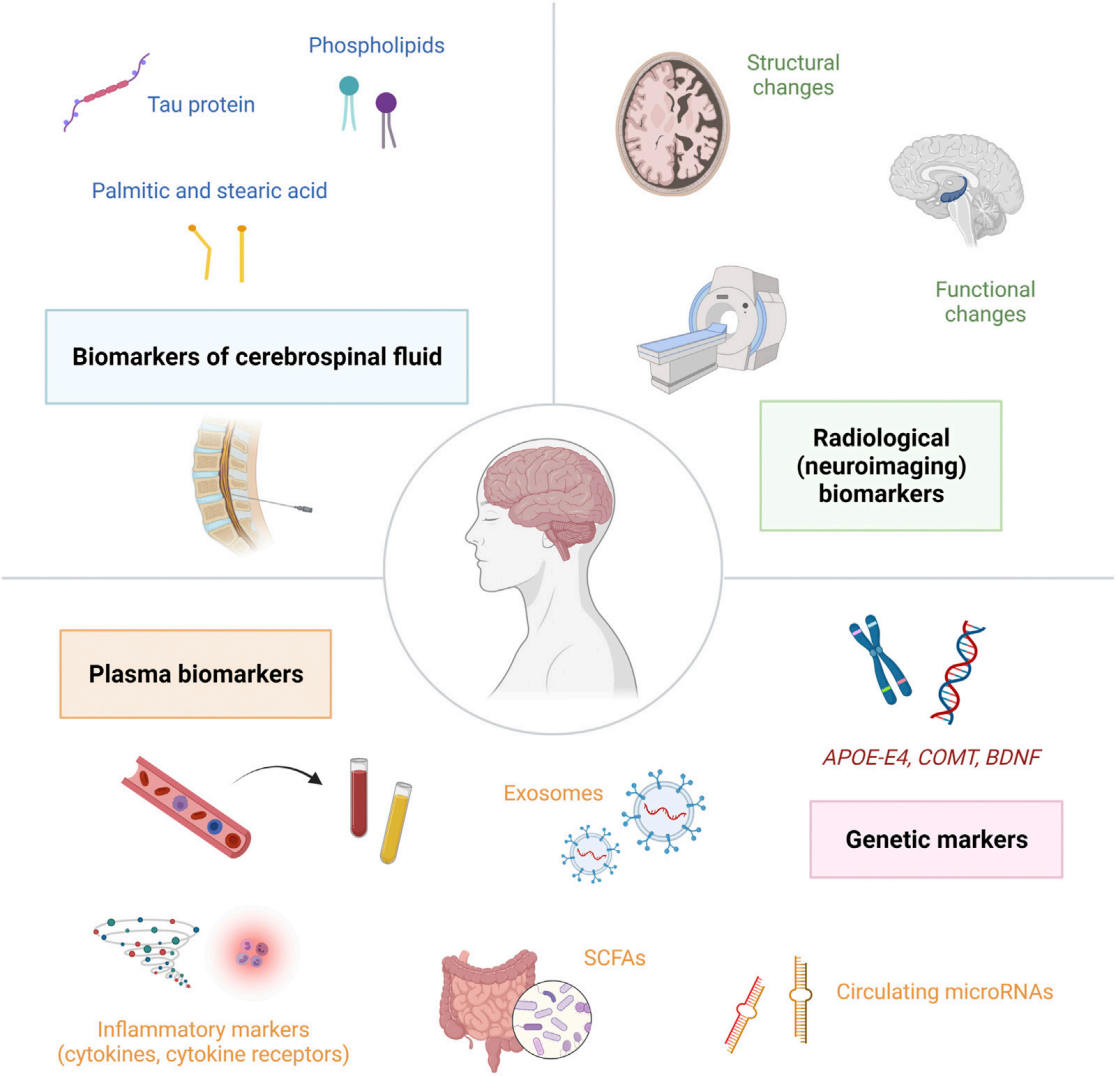
Biomarkers of cognitive dysfunction in cancer patients can be divided into several categories: genetic biomarkers, plasma biomarkers, biomarkers of cerebrospinal fluid and radiological (neuroimaging) biomarkers.; *Abbreviations:* APOE-E4 = apolipoprotein E4; BDNF = brain-derived neurotrophic factor; COMT = catechol-O-methyltransferase; SCFAs = short-chain fatty acids.

In order to identify cancer patients at increased risk for the development of CRCI or to detect cancer patients that have already developed CRCI, it is crucial to discover potential markers of cognitive impairment. Biomarkers of cognitive dysfunction in cancer patients can be divided into several categories: genetic biomarkers, plasma biomarkers, biomarkers of cerebrospinal fluid and radiological (neuroimaging) biomarkers **(** Figure 3 **)**. The molecular biomarkers are summarized in Table 2. Currently, the increased interest is focused on plasma biomarkers, that can be relatively easily measured by blood sampling before, during and after cancer treatment.

**TABLE 2:** Molecular biomarkers of CRCI with potential mechanisms of the development of cognitive dysfunction in cancer patients.

Molecular biomarkers of CRCI	Association with cognitive impairment	Potential mechanisms
**GENETIC BIOMARKERS**		
*APOE-e4* Ahles et al. (2003); Ahles et al. (2014); Amidi et al. (2017a)	worse visual memory (*p* < 0.03) and spatial ability (*p* < 0.05) Ahles et al. (2003)	Impaired neuronal repair and plasticity
	prefrontal grey matter reductions (*p* = 0.03) Amidi et al. (2017a)	Deficit in nicotinic receptor binding sites, Reduced dopaminergic activity
*COMT* Small et al. (2011); Cheng et al. (2016); (*Val allele*)	poorer attention (*p* = 0.015), verbal fluency (*p* = 0.009) and motor speed (*p* = 0.033) Small et al. (2011)	Increased metabolic degradation of dopamine resulting in its reduced amount in the frontal cortex
*BDNF* Erickson et al. (2010); Ng et al. (2016); (reduced levels)	worse spatial memory (*p* < 0.05) hippocampal volume decline (*p* < 0.001) (Erickson et al. (2010)	Impaired neurogenesis and synaptic plasticity in the dentate gyrus
**PLASMA BIOMARKERS**		
Cytokines and cytokine receptors Meyers et al. (2005); Ishikawa et al. (2012); Kesler et al. (2013b); Cheung et al. (2015)		Induction and potentiation of immune response leading to chronic inflammation.
IL-1	poorer speed performance (*p* = 0.023) Cheung et al. (2015)	Immune cells and inflammatory components crossing the blood–brain barrier causing neuroinflammation and damage of brain tissue
IL-6	worse executive function Meyers et al. (2005)	
IL-8	better memory performance Meyers et al. (2005)	
TNFRII	increased memory complaints (*p* < 0.0001) Ganz et al. 2013a)	—
Peripheral amyloid beta and tau Henneghan et al. (2020)	poorer neuropsychological tests score (*p* < 0.0001)	Increased neurodegeneration
pNF-H Natori et al. (2015)	more self-perceived cognitive symptoms (*p* = 0.77)	Axonal damage
miRNAs Sheinerman et al. (2012); Grasso et al. (2014); Xie et al. (2015); Xie et al. (2017); Kenny et al. (2019)		
miRNA-206	higher risk of MCI (all *p* < 0.05) Xie et al. (2015); Xie et al. (2017); Kenny et al. (2019) and conversion to AD (*p* = 0.0079) (Kenny et al. (2019)	Impaired synaptic plasticity and neurogenesis
miRNA-132 family	higher risk of MCI (*p* < 0.001) Sheinerman et al. (2012); Xie et al. (2015)	Silencing/down-regulation of *BDNF*
miRNA-134 family	higher risk of MCI (*p* < 0.001) Sheinerman et al. (2012)	
Exosomes Koh et al. (2020)	*under investigation*	Crossing the blood–brain barrier and changing microenvironment of the brain
SCFAs Erny et al. (2015); Ciernikova et al. (2021)	microglia defects in mice Erny et al. (2015)	Impaired microglia maturation and function
		Neuroinflammation
**BIOMARKERS OF CEREBROSPINAL FLUID**		
Palmitic and stearic acid (monounsaturated to saturated ratio) Moore et al. (2008)	declines in verbal IQ (*p* = 0.001), full-scale IQ (*p* = 0.008) and math calculation scores (*p* = 0.02)	Increased permeability of blood– brain barrier
		Impaired cell membrane integrity
Phospholipids (higher SM and lower LPC) Krull et al. (2013)	SM and reduced motor speed (from *p* < 0.001 to *p* = 0.02)	Cellular membrane and myelin defects (disrupted myelin development and demyelination)
	LPC and lower verbal working memory (*p* = 0.007)	
Tau protein Protas et al. (2009)	lower verbal IQ (*p* < 0.006)	Increased neurodegeneration

Abbreviations AD = Alzheimer’s disease; APOE-E4 = apolipoprotein E4; BDNF = brain-derived neurotrophic factor; COMT = catechol-O-methyltransferase, IL-1 = interleukin 1; IL-6 = interleukin 6; IL-8 = interleukin 8; IQ = intelligence quotient; LPC = lysophosphatidylcholine; MCI = mild cognitive impairment; pNF-H = phosphorylated neurofilament subunit H; SCFAs = short-chain fatty acids; SM = sphingomyelin; TNFRII = tumor necrosis factor receptor type II; Val = valine.

### Genetic Biomarkers

*APOE* (Apolipoprotein E) facilitates transport of lipids through bloodstream and plays an important role in neuronal plasticity and repair. *APOE-E4* gene polymorphism has been associated with cognitive impairment in Alzheimer’s disease (van der Flier et al., 2006), as well as higher risk of cognitive decline in normal aging (Schiepers et al., 2012). The association between *APOE-E4* allelic variant and CRCI was firstly demonstrated by Ahles et al. (Ahles et al., 2003) in long-term survivors of breast cancer (*N* = 51, age = 55.9 ± 8.8) and lymphoma (*N* = 29, age = 55.8 ± 11.6) treated with standard-dose chemotherapy. The mean follow-up period was 8.8 ± 4.3 years. Survivors with at least *e4* allele (*N* = 17; 21%) scored significantly lower than survivors without *e4* allele in the visual memory (mean ± SD; - 0.30 ± 1.12 vs. 0.04 ± 0.81; *p* < 0.03) and the spatial ability (mean ± SD; -0.38 ± 1.17 vs. -0.13 ± 0.97; *p* < 0.05) domains. More recent prospective study by the same authors (Ahles et al., 2014) examined the association between post-treatment cognitive changes, APOE status and smoking history in breast cancer patients treated with adjuvant therapy. Patients treated with chemotherapy (*N* = 55, age = 51.9 ± 7.1) were evaluated with neuropsychological tests prior to chemotherapy and 1-, 6- and 18-months post-chemotherapy. No chemotherapy group treated primarily with endocrine therapy (*N* = 68, age = 56.8 ± 8.3) and healthy controls (*N* = 43, age = 53.0 ± 10.1) were evaluated at similar intervals. This study found that negative effect of *APOE-E4* genotype on post-treatment cognitive functions (especially processing speed) was moderated by smoking history in both chemotherapy and endocrine therapy group. Amidi et al. (2017a) investigated cognitive changes and brain grey matter morphology in testicular cancer patients (*N* = 65) with stage I-III undergoing treatment, as well as associations with genotype, immune and endocrine markers. Twenty-two patients received chemotherapy (+CT) and 43 did not (−CT). Patients were assessed (neuropsychological testing, whole-brain MRI and blood samples) after surgery prior to further treatment and 6 months after. Both groups showed higher proportions of patients with cognitive decline compared to healthy controls (63.6% in +CT group and 39.5% in -CT group vs. 8% in HC; *p* < 0.05). MRI revealed widespread grey matter reductions in both patient groups, with prefrontal reductions specific to the +CT group (*p* = 0.02). Worse cognitive performance was associated with prefrontal grey matter reductions in the +CT patients (*r* = −0.49, *p* = 0.03), but not with global grey matter reductions (*r* = −0.23, *p* = 0.33). In addition, the study revealed a significant interaction effect between *APOE-E4* status and chemotherapy on cognition [F (1,46) = 4.50, *p* = 0.03] with poorer cognitive performance of APOE *e4* allele carriers.

*COMT* (Catechol-O-methyltransferase) regulates the metabolic degradation of catecholamines through methylation of dopamine, influencing its levels in the prefrontal cortex. Small et al. (2011) evaluated the COMT *Val158Met* single-nucleotide polymorphism in breast cancer patients treated with radiotherapy (*N* = 58), and/or chemotherapy (*N* = 72). The presence of the Val allele of COMT is related to higher enzymatic activity, leading to more degradation of dopamine and decrease of its availability at synaptic receptors. This study found that patients, who were *COMT-Val* carriers had worse performance on tests of attention (*p* = 0.015), verbal fluency (*p* = 0.009), and motor speed (*p* = 0.033) compared to *COMT-Met* homozygotes. In a study by Cheng et al. (Cheng et al., 2016) the presence of *rs165599* polymorphism in COMT gene was associated with impaired retrospective memory in breast cancer patients (*N* = 245; triple-negative, TNBC = 80; non-triple negative, NTNBC = 165) treated with standard-dose adjuvant chemotherapy, but no hormonal therapy. Cognitive scores of breast cancer patients after chemotherapy were significantly worse (higher) compared to those before chemotherapy (17.21 ± 4.59 vs. 16.23 ± 4.03; *p* < 0.01) and TNBC patients scored poorer than NTNBC patients after chemotherapy (19.10 ± 2.36 vs. 16.29 ± 5.10; *p* < 0.01).

*BDNF* (Brain-derived neurotrophic factor) found mainly in the hippocampus, caudate nucleus and prefrontal cortex is involved in neurogenesis and synaptic plasticity. Erickson et al. (2010) studied relation between serum levels of BDNF, changes in hippocampal volume and memory deficits in adults (*N* = 142) without dementia between 59 and 81 years of age. This study found increasing age was associated with decline in hippocampal volumes on MRI (*p* < 0.001), reduced levels of serum BDNF and poorer memory performance (evaluated with spatial memory task in which memory load was parametrically increased). A study by Ng et al. (2016) evaluated effect of *Val66Met* polymorphism (*rs6265*) in BDNF gene on CRCI in patients receiving adjuvant chemotherapy for early-stage breast cancer (*N* = 145; mean age = 50.8 ± 8.8; 82.1% were of Asian ethnicity). Patients were assessed prospectively prior to treatment, 6 weeks after the start of treatment and 3 months after the start of treatment (at the end of chemotherapy). Interestingly, *Met/Met* genotype was associated with statistically significant lower odds of developing CRCI (OR = 0.26; 95% CI: 0.08–0.92; *p* < 0.036). The carriers of *Met* allele were less likely to experience impairment in the domains of verbal fluency (OR = 0.34; 95% CI: 0.12–0.90; *p* < 0.031) and multitasking ability (OR = 0.37; 95% CI: 0.15–0.91; *p* < 0.030) in comparison with the *Val/Val* homozygotes, suggesting protective effect of *Met* allele against CRCI.

Additionally, *rs949963* polymorphism of interleukin 1 receptor, type 1 (IL1R1) gene was associated with lower perceived attentional function in breast cancer patients (*N* = 397; OR = 1.98; 95% CI: 1.18, 3.30; *p* = 0.009), suggesting that cytokine dysregulation may negatively impact some cognitive domains (Merriman et al., 2014). A different gene expression with perturbed inflammatory pathways of cytokine-cytokine receptor interaction, mTOR, MAPK, IL-17 and TNF were also described to be associated with low levels of cognitive functions in cancer patients (all *p* < 0.05) (Oppegaard et al., 2021).

### Plasma Biomarkers

An important group of plasma biomarkers associated with CRCI represent inflammatory markers, mainly cytokines (IL-1, IL-6, TNF-alpha), cytokine receptors (sTNFRI, sTNFRII) and other inflammatory components. Cytokines are small secreted proteins produced by immune cells in order to regulate and influence immune response (Takeuchi and Akira, 2010). Pro-inflammatory cytokines, including IL-1, IL-6, IL-8, TNF-alpha, can induce or potentiate inflammation through various pathways. IL-1 (interleukin 1) family of cytokines comprises 11 proteins, that are major mediators of innate immune reactions by activation of monocytes/macrophages and T-cell proliferation, and play an important role in autoinflammatory diseases (Weber et al., 2010). IL-6 (interleukin 6) is involved in the acute phase response, final maturation of B cells into antibody-producing cells, macrophage differentiation and T-cell differentiation towards T-helper 2 (Th2) cells and Th17 cells (Diehl and Rincon, 2002). IL-8 (interleukin 8) is a chemokine for granulocytes, primarily neutrophils. It is produced by macrophages, but also endothelial cells and smooth muscle cells. IL-8 guides neutrophils to the direction of inflammation (chemotaxis) and stimulates phagocytosis (Baggiolini and Clark-Lewis, 1992). TNF-alpha (tumor necrosis factor alpha) is produced by monocytes/macrophages during acute phase of inflammation and is responsible for numerous signalling events within cells, leading to programmed cell death or necrosis. Levels of cytokines and their receptors are often increased in cancer patients and could be determinants of CRCI (Meyers et al., 2005; Ishikawa et al., 2012; Kesler et al., 2013b; Cheung et al., 2015; Lyon et al., 2016; Williams et al., 2018). Meyers et al. (2005) evaluated levels of cytokines and presence of cognitive impairment and fatigue in patients with acute myelogenous leukemia (AML) and myelodysplastic syndrome (MDS) prior to chemotherapy. Levels of IL-1, IL-1RA, IL-6, IL-8, and TNF-alpha were highly elevated compared to healthy controls. Higher IL-6 levels were associated with poorer executive function, but higher IL-8 levels were associated with better memory performance. IL-6, IL-1RA and TNF-alpha levels were related to fatigue. A study by Cheung et al. (2015) found that higher levels of IL-1β and IL-6 were associated with more severe cognitive disturbances, specifically IL-1β levels were associated with poorer response speed performance. However, elevated IL-4 levels were associated with better response speed performance and fewer cognitive complaints in breast cancer patients, suggesting protective role of this cytokine against CRCI. Ganz et al. (2013a) showed that levels of TNF receptor type II (TNFRII) were significantly higher in chemotherapy-treated patients compared to controls, with no differences observed in IL-1RA, IL-6 or CRP. In addition, there was a significant correlation between plasma TNFRII and self-reported memory complaints. Another study found elevated IL-6 and TNF-α levels after approximately 5 years after the completion of chemotherapy of breast cancer (Kesler et al., 2013b). These findings were supported by Janelesins et al. (2012), showing different inflammatory responses induced by different chemotherapy regimens. Interestingly, a study by van der Willik et al. (2018) showed cancer survivors had increased levels of inflammation on average 20 years after treatment and that they were associated with lower cognitive performance. The chronic inflammation induced by cancer and cancer treatments is associated with higher levels of pro-inflammatory cytokines and cytokine receptors in cancer patients. Furthermore, if the permeability of blood-brain barrier is impaired, inflammatory components may reach nervous tissue and possibly cause damage to neural cells, leading to cognitive dysfunction.

A recent study by Henneghan et al. (2020) evaluated peripheral amyloid beta (Aβ) and tau, biomarkers of neurodegeneration, and cytokines, in relation to cognitive functions in breast cancer survivors, suggesting potential interplay between neurodegenerative biomarkers and cytokines to influence cognitive functioning of these patients.

Another possible plasma biomarker of CRCI is axonal phosphorylated neurofilament subunit H (pNF-H). Its levels are increased in the blood of patients who have had acute brain ischemic stroke and associated with the severity of the stroke (Singh et al., 2011). Therefore Natori et al. (2015) measured pNF-H levels and cognitive changes in breast cancer patients undergoing chemotherapy, showing increased serum pNF-H in a cumulative dose-dependent manner and suggesting it as biomarker of neural damage after chemotherapy. However, cognitive failure questionnaires used in this study did not show significant cognitive impairment (*p* = 0.77).

Circulating microRNAs (miRNAs), a family of short single-stranded non-coding RNAs of 21–22 nucleotides in length, making up about 1% of the human genome, could also be potential biomarkers of CRCI. The main function of miRNAs is modulation of gene expression at the post-transcriptional level, causing inhibition of translation of mRNA or its degradation (Piscopo et al., 2019). Some miRNAs act as important regulators of various biological functions in the brain, including synaptic plasticity and neurogenesis, and may indirectly affect neurogenesis by regulating neural stem cell proliferation and repair. For this reason, they play an important role in the development of neurodegenerative diseases and other conditions in which impaired brain function is present (Grasso et al., 2014). Studies evaluating levels of expression of certain miRNAs are primarily focused on the diagnosis of mild cognitive impairment (MCI) as an intermediate stage between normal cognition and dementia, in order to identify a potential biomarker of MCI and its progression to Alzheimer’s disease. The most important microRNAs that have been shown to associate with MCI include miRNA-206, miRNA-132, and miRNA-134. These miRNAs are overexpressed in patients with MCI and even higher levels correlate with the rate of decline in cognitive functions. Their target genes are *BDNF* and *SIRT1*, which are involved in cognitive processes in the brain and their expression is reduced in patients with MCI. Several studies suggest that the detection of these microRNAs could serve for the early diagnosis of MCI (Sheinerman et al., 2012; Xie et al., 2015; Xie et al., 2017; Kenny et al., 2019). Even though an association between these miRNAs and CRCI has not been demonstrated in clinical studies yet, it may be interesting subject of future research.

The role of exosomes in cancer has been well-known (Zhang et al., 2015; Han et al., 2019), but cancer exosomes and their interactions with the nervous system to modulate various neurological processes are under active investigation. Exosomes are small endocytic vesicles formed by the inward budding of multivesicular bodies, released into the extracellular environment through exocytic pathway (Rajagopal and Harikumar, 2018). They can influence various neurological processes such as neurogenesis, synaptic plasticity, neuronal stress response and cell-to-cell communication, suggesting their potential role in the development of CRCI by multiple studies (Koh et al., 2020).

A production of short-chain fatty acids (SCFAs), that are likely to play a key role in neuro-immuno-endocrine regulation (Silva et al., 2020), might be a mechanism by which gut microbiome influences brain function. SCFAs are small organic monocarboxylic acids with a chain length of up to 6 carbon atoms. Acetate (C2), propionate (C3) and butyrate (C4) are the major metabolites produced in the colon by bacterial fermentation of indigestible polysaccharides such as fiber and resistant starch (Pascale et al., 2018). At the same time, bacterial production of SCFAs has been shown to control the maturation of the microglia and affect its functions. The absence or alteration of a healthy gut microbiome causes impaired microglia development, leading to disruption of innate immune responses. Additionally, application of a mixture of the three major SCFAs led to microglia maturation and restoration in germ-free mice (Erny et al., 2015).

### Biomarkers of Cerebrospinal Fluid

Analysis of CSF can provide promising information about biological processes in the CNS and possible cognitive alterations in cancer patients, even though lumbar puncture as a sampling method is more invasive. Moore et al. (2008) evaluated levels of palmitic and stearic acid (ratio between monounsaturated/saturated) in the CSF of pediatric patients with acute lymphoblastic leukemia (ALL) treated with methotrexate for more than 3 years. Results showed a significant increase in the ratio of monounsaturation to saturation of both fatty acids, positively correlating with the number of intrathecal methotrexate doses received during the first year, and negatively correlating with decreased global intelligence ability. Krull et al. (2013) studied associations between changes in CSF phospholipids and neurocognitive function in children undergoing chemotherapy for ALL. Sphingomyelin (SM) and lysophosphatidylcholine (LPC) concentrations were increased in CSF after chemotherapy induction and were associated with lower motor speed, visual and verbal working memory. A study by Protas et al. (2009) showed a significant elevated levels of tau protein in CSF after induction and during consolidation treatment with methotrexate in ALL patients, compared to baseline levels. In addition, levels of tau protein were negatively correlated with verbal abilities.

### Radiological (Neuroimaging) Biomarkers

Over the past few years, great progress has been made in neuroimaging research on cognitive functions of cancer patients. Neuroimaging studies provide important information on structural, functional and molecular changes in brain, helping to objectify the presence of cognitive impairment. Structural magnetic resonance imaging (MRI), functional MRI (fMRI), diffusion tensor imaging (DTI), MR spectroscopy (MRS), and positron emission tomography (PET) have been used in the clinical research studying the impact of cancer treatment on cognition, with the main focus on breast cancer patients treated by chemotherapy (Saykin et al., 2013). Multiple studies have reported reductions in white matter microstructure, reductions in grey matter volume or density, alterations in brain activation and connectivity after chemotherapy (Li and Caeyenberghs, 2018). Some studies showed functional hyperactivation and hyperconnectivity of brain regions after cancer treatment, but it could be interpreted as compensatory mechanisms against brain injury (Menning et al., 2015; Apple et al., 2018). A study by Amidi et al. (2017b) evaluated early effects of BEP chemotherapy on brain structure in testicular cancer patients, finding multiple reductions in grey matter density and especially reductions in prefrontal areas were associated with cognitive decline. Koppelmans et al. (2012b) evaluated breast cancer survivors 20 years after chemotherapy and showed global reductions in grey matter volume associated with worse performance on neuropsychological tests. More other studies support these findings and also showed structural and functional brain changes in patients with cognitive dysfunction related to cancer treatment (Deprez et al., 2012; McDonald et al., 2012; Correa et al., 2013; Lopez Zunini et al., 2013; McDonald et al., 2013; Stouten-Kemperman et al., 2015).

## Treatment of Cancer-Related Cognitive Impairment

While researchers and clinicians are intensively studying the risk factors, mechanisms and biomarkers of CRCI development, the increasing number of studies are evaluating potential methods for effective treatment of cognitive dysfunction in cancer patients and survivors. A variety of therapeutical interventions has been tested to reduce self-reported cognitive symptoms and restore CRCI. These interventions include cognitive rehabilitation/training, physical activity, mind-body interventions and pharmacotherapy.

### Cognitive Rehabilitation/Training

Cognitive rehabilitation refers to behaviorally orientated interventions, which are designed to improve cognitive performance and to manage/compensate for cognitive deficits. Cognitive training involves regular practice of skills in order to restore attention, psychomotor speed, memory and executive functioning. A study in early-stage breast cancer survivors with cognitive complaints (*N* = 82; mean age = 56.5 ± 8.5; time post-treatment = 5.5 ± 4.2 years) reported improvements in objectively measured memory and speed of processing, as well as perceived cognitive functioning immediately after in-person cognitive training delivered in a group setting and at 2-months follow-up, compared to waitlist controls (all *p* < 0.04) (Von Ah et al., 2012). Several other studies in breast cancer survivors showed similar results, some of them with home-based training intervention (Kesler et al., 2013a; Ercoli et al., 2015; Ferguson et al., 2016; Park et al., 2017; Meneses et al., 2018). A study evaluating web-based cognitive training in breast cancer survivors with CRCI (*N* = 94; mean age = 54.98 ± 8.51) did not result in improvement of perceived cognitive functioning, but improved performance was observed on verbal learning and working memory tests at 5-months follow-up, compared to waitlist controls (*p* = 0.040–0.043) (Damholdt et al., 2016). Bray et al. (2017) randomized solid tumor cancer survivors (*N* = 242; mean age = 53 years; 95% female) with cognitive symptoms 6–60 months after adjuvant chemotherapy to a home-based cognitive training intervention or to a standard care (authors did not specify). Cognitive functions were assessed with FACT-Cog questionnaires and neuropsychological tests after the intervention and 6 months later. This study showed significant improvement in perceived cognitive impairment, lower levels of anxiety/depression and fatigue in the intervention group at both assessment times, but no significant differences between the groups on neuropsychological testing (Bray et al., 2017). Recent systematic reviews on cognitive rehabilitation and training in cancer patients and survivors with cognitive problems showed that available evidence support clinical implementation of these interventions for treatment of CRCI. However, more clinical trials are needed to assess the effectiveness, optimal dose, delivery, access and cost of different cognitive programs (Fernandes et al., 2019; Von Ah and Crouch, 2020).

### Physical Activity

Physical activity has been shown to decrease a variety of cancer- and cancer treatment-related physiological and psychological symptoms and to be beneficial for cognitive functions in general. The important role of exercise was also enhanced by its implementation in the guidelines for cancer survivors (Schmitz et al., 2010; Campbell et al., 2019). In animal models, physical exercise reduced chemotherapy-related cognitive impairment by preventing suppression of hippocampal neurogenesis (Winocur et al., 2014). Multiple clinical studies investigated different types of exercise interventions (e.g., aerobic, resistance, mixed, yoga, etc.) in cancer patients during or after the end of treatment and showed improvement in self-reported cognitive functioning and neuropsychological performance, although many studies have evaluated effects on CRCI as a secondary outcome (Campbell et al., 2020). A study in breast cancer survivors (*N* = 317; mean age = 59.1 ± 7.9; stage 0—IIIc; all less than 10 years post-treatment) showed moderate and vigorous levels of physical activity moderated breast cancer treatment effects on depression and cognition. Furthermore, the effects of exercise could be partially explained by changes in depressive symptoms (Bedillion et al., 2019). Our recent study in testicular cancer patients also showed regular exercise had positive effects on different physical and psychosocial outcomes during and after cancer treatment, including cognitive impairment (Amiri et al., 2021). Therefore, the current evidence supported physical activity as a potential management strategy for CRCI, although the questions of optimal timing, duration, mode or intensity of the exercise should be answered in future research and individualized for patients based on their psychosocial and physiological limitations and preferences.

### Mind-Body Interventions

Mind-body interventions are supposed to bring an awareness of patient’s individual potential for healing or restoration and through this to improve cognitive function as well. These interventions include meditation (Milbury et al., 2013), guided imagery (Freeman et al., 2015), mindfulness-based stress reduction (MBSR) (Hoffman et al., 2012; Johns et al., 2016), restorative environment (Cimprich and Ronis, 2003) and biofeedback (Alvarez et al., 2013). For example, meditation in breast cancer patients has been showed to improve verbal memory, short-term memory and processing speed (Milbury et al., 2013). In addition, EEG biofeedback in breast cancer survivors had a potential for reducing negative cognitive and emotional symptoms of cancer treatment with improving fatigue and sleep patterns (Alvarez et al., 2013).

### Pharmacotherapy

The National Comprehensive Cancer Network (NCCN) recommended “the use of nonpharmacologic interventions for CRCI whenever possible, with pharmacologic interventions as a last line of therapy in cancer survivors for whom other interventions have been insufficient” (Denlinger et al., 2014). Many pharmacotherapeutic interventions for the management of CRCI in non-CNS cancer patients have been tested in clinical studies. However, the results have been conflicting and no uniform guidelines for pharmacological prevention or treatment of CRCI have yet been established (Karschnia et al., 2019). Pharmacotherapies evaluated as a potential management for CRCI include CNS stimulants (e.g., methylphenidate and modafinil), anti-dementia drugs (e.g., donepezil and memantine), selective-serotonin reuptake inhibitors (e.g., sertraline and paroxetine) and other agents, including Ginkgo biloba and erythropoietin. Clinical trials assessing the effects of methylphenidate have reported mixed results (Mar Fan et al., 2008; Lower et al., 2009) with one study showing improvements in cognition and ability to work more hours (Escalante et al., 2014). Modafinil was reported to improve memory and attention skills in breast cancer survivors (Kohli et al., 2009), as well as psychomotor speed and attention in patients with multiple advanced cancers (Lundorff et al., 2009). Donepezil was associated with better cognitive performance in multiple memory tasks in breast cancer patients 1–5 years after receiving adjuvant chemotherapy (Lawrence et al., 2016). Antidepressant agent sertraline improved executive functions and quality of life in patients with advanced cancer (Li et al., 2014). Barton et al. (2013) in their clinical trial evaluated Ginkgo biloba for the prevention of CRCI in patients with breast cancer receiving adjuvant chemotherapy and did not show any effect on preventing cognitive dysfunction. On the contrary, Chang et al. (2004) reported improvement of cognition and functional well-being with the use of epoetin alfa in breast cancer treated with adjuvant chemotherapy. Furthermore, novel treatment strategies for cognitive dysfunction in cancer survivors are emerging with an increasing knowledge on the underlying molecular mechanisms.

## Future Directions and Recommendations

Long-term cognitive impairment is an emerging health issue in survivors of cancer. While evidence supporting the increased cognitive difficulties in long-term survivors of cancers is emerging, the results of available studies show certain level of inconsistence and controversies.

Relevant standardized tools with high sensitivity and reliability for the assessment of CRCI still need to be established. In addition, the discordance between the self-reported and objectively assessed cognitive function may be difficult to explain and should serve as a substrate for scientific exploration. Furthermore, we believe that self-reported cognitive impairment should not be disregarded and should be carefully assessed and managed by the attending clinician.

The impact of CRCI on patients’ quality of life, as well as on socioeconomics in the population with growing number of cancer survivors, remains serious issue to discuss and investigate. Actually, negative effect of even mild cognitive impairment may be potentiated by psychological factors (such as anxiety, depression or fatigue), leading to significant self-perceived cognitive changes. In addition, these cognitive difficulties may affect different aspects of everyday life (e.g., family, work and social life).

Awareness of CRCI among clinicians is essential in order to prevent, diagnose and manage cognitive impairment appropriately. However, recommendations for prevention, diagnostics and management of CRCI from leading cancer organizations are in demand. Clinicians thinking of CRCI as a side effect of cancer treatment may consider risk-benefit of certain therapy to avoid overtreatment. Clinicians should also advocate for maintaining the healthy lifestyle with regular physical activity, balanced diet and smoking cessation. The emphasis should be placed on mental health as well, even if psychological intervention is needed. Another important issue for clinicians is their ability to adequately communicate the possible side effects of cancer treatment to the patients (especially vulnerable individuals) without causing “unnecessary toxicity” of incorrect communication.

The increasing number of cancer survivors provides a strong rationale to systematic establishment of specialized survivorship clinics, where diagnostics and management of late toxicities of cancer and cancer treatment should be implemented. Furthermore, comprehensive assessment of quality of life should be performed in all survivors of cancer and we advocate for life-long annual follow-up performed by a clinician trained in cancer survivorship.

Future research should focus on the understanding of underlying molecular mechanisms of CRCI. In addition, clinical studies should also implement possible novel preventive and treatment strategies (e.g., educational, psychological, exercise, pharmacologic, etc.). Discovery of novel biomarkers is crucial for identification of early CRCI onset or uncovering the population in risk for CRCI development. This could possibly help clinicians to design and implement early treatment interventions in order to lower the burden of cognitive changes on the long-term quality of life.

## Conclusion

This review summarizes the current evidence on cancer-related cognitive impairment, that is experienced by many cancer survivors with a significant impact on their everyday life. The research has been focused mainly on breast cancer patients, but CRCI has been reported in multiple types of cancer, including colorectal, ovarian, testicular cancer, etc. Incidence of CRCI is associated with radiotherapy, chemotherapy, hormonal therapy and even novel systemic therapies. However, CRCI has been also detected prior to cancer treatment, suggesting possible negative effects of cancer itself with or without the psychological distress in its pathogenesis. Various mechanisms are suggested to be responsible for CRCI, including direct neurotoxic injury of systemic treatment and radiation, as well as chronic neuroinflammation, that is very likely to be the central mechanism of CRCI pathogenesis. There is increasing evidence of potential genetic (*APOE, COMT, BDNF*), plasma (e.g., inflammatory molecules, circulating microRNAs, exosomes, short-chain fatty acids and others), cerebrospinal fluid and neuroimaging biomarkers of CRCI in cancer patients. Various therapeutical interventions have been tested and shown to alleviate or restore CRCI, including cognitive rehabilitation/training, physical activity, mind-body interventions and several pharmacological agents. Cognitive dysfunction may represent a serious issue in the increasing population of cancer survivors and therefore intensive research is necessary in order to develop appropriate prevention, diagnostics and management of CRCI.

## References

[B1] Aaronson N. K. Ahmedzai S. Bergman B. Bullinger M. Cull A. Duez N. J. (1993). The European Organization for Research and Treatment of Cancer QLQ-C30: a Quality-Of-Life Instrument for Use in International Clinical Trials in Oncology. JNCI J. Natl. Cancer Inst. 85 (5), 365–376. 10.1093/jnci/85.5.365 8433390

[B2] Ahles T. A. Li Y. McDonald B. C. Schwartz G. N. Kaufman P. A. Tsongalis G. J. (2014). Longitudinal Assessment of Cognitive Changes Associated with Adjuvant Treatment for Breast Cancer: the Impact of APOE and Smoking. Psycho-Oncology 23 (12), 1382–1390. 10.1002/pon.3545 24789331 PMC4214914

[B3] Ahles T. A. Root J. C. (2018). Cognitive Effects of Cancer and Cancer Treatments. Annu. Rev. Clin. Psychol. 14, 425–451. 10.1146/annurev-clinpsy-050817-084903 29345974 PMC9118140

[B4] Ahles T. A. Saykin A. J. (2007). Candidate Mechanisms for Chemotherapy-Induced Cognitive Changes. Nat. Rev. Cancer 7 (3), 192–201. 10.1038/nrc2073 17318212 PMC3329763

[B5] Ahles T. A. Saykin A. J. McDonald B. C. Furstenberg C. T. Cole B. F. Hanscom B. S. (2008). Cognitive Function in Breast Cancer Patients Prior to Adjuvant Treatment. Breast Cancer Res. Treat. 110 (1), 143–152. 10.1007/s10549-007-9686-5 17674194 PMC3114441

[B6] Ahles T. A. Saykin A. J. McDonald B. C. Li Y. Furstenberg C. T. Hanscom B. S. (2010). Longitudinal Assessment of Cognitive Changes Associated with Adjuvant Treatment for Breast Cancer: Impact of Age and Cognitive reserve. Jco 28 (29), 4434–4440. 10.1200/JCO.2009.27.0827 PMC2988635 20837957

[B7] Ahles T. A. Saykin A. J. Noll W. W. Furstenberg C. T. Guerin S. Cole B. (2003). The Relationship of APOE Genotype to Neuropsychological Performance in Long-Term Cancer Survivors Treated with Standard Dose Chemotherapy. Psycho-Oncology 12 (6), 612–619. 10.1002/pon.742 12923801

[B8] Alibhai S. M. H. Timilshina N. Duff-Canning S. Breunis H. Tannock I. F. Naglie G. (2017). Effects of Long-Term Androgen Deprivation Therapy on Cognitive Function over 36 Months in Men with Prostate Cancer. Cancer 123 (2), 237–244. 10.1002/cncr.30320 27583806

[B9] Allen B. D. Apodaca L. A. Syage A. R. Markarian M. Baddour A. A. D. Minasyan H. (2019). Attenuation of Neuroinflammation Reverses Adriamycin-Induced Cognitive Impairments. Acta Neuropathol. Commun. 7 (1), 186. 10.1186/s40478-019-0838-8 31753024 PMC6868786

[B10] Alvarez J. Meyer F. L. Granoff D. L. Lundy A. (2013). The Effect of EEG Biofeedback on Reducing Postcancer Cognitive Impairment. Integr. Cancer Ther. 12 (6), 475–487. 10.1177/1534735413477192 23584550

[B11] Amidi A. Agerbæk M. Wu L. M. Pedersen A. D. Mehlsen M. Clausen C. R. (2017a). Changes in Cognitive Functions and Cerebral Grey Matter and Their Associations with Inflammatory Markers, Endocrine Markers, and APOE Genotypes in Testicular Cancer Patients Undergoing Treatment. Brain Imaging Behav. 11 (3), 769–783. 10.1007/s11682-016-9552-3 27240852

[B12] Amidi A. Christensen S. Mehlsen M. Jensen A. B. Pedersen A. D. Zachariae R. (2015a). Long-term Subjective Cognitive Functioning Following Adjuvant Systemic Treatment: 7-9 Years Follow-Up of a Nationwide Cohort of Women Treated for Primary Breast Cancer. Br. J. Cancer 113 (5), 794–801. 10.1038/bjc.2015.243 26171932 PMC4559822

[B13] Amidi A. Hosseini S. M. H. Leemans A. Kesler S. R. Agerbæk M. Wu L. M. (2017b). Changes in Brain Structural Networks and Cognitive Functions in Testicular Cancer Patients Receiving Cisplatin-Based Chemotherapy. J. Natl. Cancer Inst. 109 (12). 10.1093/jnci/djx085 29617869

[B14] Amidi A. Wu L. M. Pedersen A. D. Mehlsen M. Pedersen C. G. Rossen P. (2015b). Cognitive Impairment in Testicular Cancer Survivors 2 to 7 Years after Treatment. Support Care Cancer 23 (10), 2973–2979. 10.1007/s00520-015-2663-3 25716340

[B15] Amiri A. Chovanec M. Oliva V. Sedliak M. Mego M. Ukropec J. (2021). Chemotherapy‐induced Toxicity in Patients with Testicular Germ Cell Tumors: The Impact of Physical Fitness and Regular Exercise. Andrology 2021, 1–14. 10.1111/andr.13078 34245663

[B16] Andreotti C. Root J. C. Ahles T. A. McEwen B. S. Compas B. E. (2015). Cancer, Coping, and Cognition: a Model for the Role of Stress Reactivity in Cancer-Related Cognitive Decline. Psycho-Oncology 24 (6), 617–623. 10.1002/pon.3683 25286084 PMC4387099

[B17] Anstey K. J. Sargent-Cox K. Cherbuin N. Sachdev P. S. (2015). Self-Reported History of Chemotherapy and Cognitive Decline in Adults Aged 60 and Older: The PATH through Life Project. Gerona 70 (6), 729–735. 10.1093/gerona/glt195 24368774

[B18] Apple A. C. Schroeder M. P. Ryals A. J. Wagner L. I. Cella D. Shih P.-A. (2018). Hippocampal Functional Connectivity Is Related to Self-Reported Cognitive Concerns in Breast Cancer Patients Undergoing Adjuvant Therapy. NeuroImage: Clin. 20, 110–118. 10.1016/j.nicl.2018.07.010 30094161 PMC6077172

[B19] Baggiolini M. Clark-Lewis I. (1992). Interleukin-8, a Chemotactic and Inflammatory Cytokine. FEBS Lett. 307 (1), 97–101. 10.1016/0014-5793(92)80909-z 1639201

[B20] Bagnall-Moreau C. Chaudhry S. Salas-Ramirez K. Ahles T. Hubbard K. (2019). Chemotherapy-Induced Cognitive Impairment Is Associated with Increased Inflammation and Oxidative Damage in the Hippocampus. Mol. Neurobiol. 56 (10), 7159–7172. 10.1007/s12035-019-1589-z 30989632 PMC6728167

[B21] Baik S. H. Kury F. S. P. McDonald C. J. (2017). Risk of Alzheimer's Disease Among Senior Medicare Beneficiaries Treated with Androgen Deprivation Therapy for Prostate Cancer. Jco 35 (30), 3401–3409. 10.1200/JCO.2017.72.6109 PMC5648173 28841388

[B22] Barton D. L. Burger K. Novotny P. J. Fitch T. R. Kohli S. Soori G. (2013). The Use of Ginkgo Biloba for the Prevention of Chemotherapy-Related Cognitive Dysfunction in Women Receiving Adjuvant Treatment for Breast Cancer, N00C9. Support Care Cancer 21 (4), 1185–1192. 10.1007/s00520-012-1647-9 23150188 PMC3587364

[B23] Bedillion M. F. Ansell E. B. Thomas G. A. (2019). Cancer Treatment Effects on Cognition and Depression: The Moderating Role of Physical Activity. The Breast 44, 73–80. 10.1016/j.breast.2019.01.004 30685529

[B24] Bender C. M. Merriman J. D. Gentry A. L. Ahrendt G. M. Berga S. L. Brufsky A. M. (2015). Patterns of Change in Cognitive Function with Anastrozole Therapy. Cancer 121 (15), 2627–2636. 10.1002/cncr.29393 25906766 PMC4512875

[B25] Benedict R. H. B. Schretlen D. Groninger L. Brandt J. (1998). Hopkins Verbal Learning Test - Revised: Normative Data and Analysis of Inter-form and Test-Retest Reliability. The Clin. Neuropsychologist 12 (1), 43–55. 10.1076/clin.12.1.43.1726

[B26] Benton A. L. Hamsher D. K. Sivan A. B. (1994). Multilingual Aphasia Examination. Iowa City: AJA Associates.

[B27] Berlin C. Lange K. Lekaye H. C. Hopland K. Phillips S. Piao J. (2020). Long-term Clinically Relevant Rodent Model of Methotrexate-Induced Cognitive Impairment. Neuro Oncol. 22 (8), 1126–1137. 10.1093/neuonc/noaa086 32242229 PMC7594568

[B28] Bhojwani D. Sabin N. D. Pei D. Yang J. J. Khan R. B. Panetta J. C. (2014). Methotrexate-induced Neurotoxicity and Leukoencephalopathy in Childhood Acute Lymphoblastic Leukemia. Jco 32 (9), 949–959. 10.1200/JCO.2013.53.0808 PMC3948096 24550419

[B29] Borenstein A. R. Mortimer J. A. Wu Y. Jureidini-Webb F. M. Fallin M. D. Small B. J. (2006). Apolipoprotein E and Cognition in Community-Based Samples of African Americans and Caucasians. Ethn. Dis. 16 (1), 9–15. 16599342

[B30] Borre Y. E. O’Keeffe G. W. Clarke G. Stanton C. Dinan T. G. Cryan J. F. (2014). Microbiota and Neurodevelopmental Windows: Implications for Brain Disorders. Trends Mol. Med. 20 (9), 509–518. 10.1016/j.molmed.2014.05.002 24956966

[B31] Boscher C. Joly F. Clarisse B. Humbert X. Grellard J.-M. Binarelli G. (2020). Perceived Cognitive Impairment in Breast Cancer Survivors and its Relationships with Psychological Factors. Cancers 12 (10), 3000. 10.3390/cancers12103000 PMC7602817 33081111

[B32] Bray V. J. Dhillon H. M. Bell M. L. Kabourakis M. Fiero M. H. Yip D. (2017). Evaluation of a Web-Based Cognitive Rehabilitation Program in Cancer Survivors Reporting Cognitive Symptoms after Chemotherapy. Jco 35 (2), 217–225. 10.1200/JCO.2016.67.8201 28056205

[B33] Bray V. J. Dhillon H. M. Vardy J. L. (2018). Systematic Review of Self-Reported Cognitive Function in Cancer Patients Following Chemotherapy Treatment. J. Cancer Surviv 12 (4), 537–559. 10.1007/s11764-018-0692-x 29728959

[B34] Bromis K. Gkiatis K. Karanasiou I. Matsopoulos G. Karavasilis E. Papathanasiou M. (2017). Altered Brain Functional Connectivity in Small-Cell Lung Cancer Patients after Chemotherapy Treatment: A Resting-State fMRI Study. Comput. Math. Methods Med. 2017, 1–12. 10.1155/2017/1403940 PMC5535744 28798808

[B35] Buskbjerg C. R. Zachariae R. Agerbæk M. Gravholt C. H. Haldbo-Classen L. Hosseini S. M. H. (2021). Cognitive Impairment and Associations with Structural Brain Networks, Endocrine Status, and Risk Genotypes in Newly Orchiectomized Testicular Cancer Patients. Brain Imaging Behav. 10.1007/s11682-021-00492-x 34392471

[B36] Callaghan C. K. O’Mara S. M. (2015). Long-term Cognitive Dysfunction in the Rat Following Docetaxel Treatment Is Ameliorated by the Phosphodiesterase-4 Inhibitor, Rolipram. Behav. Brain Res. 290, 84–89. 10.1016/j.bbr.2015.04.044 25940764

[B37] Campbell K. L. Winters-Stone K. M. Wiskemann J. May A. M. Schwartz A. L. Courneya K. S. (2019). Exercise Guidelines for Cancer Survivors: Consensus Statement from International Multidisciplinary Roundtable. Med. Sci. Sports Exerc. 51 (11), 2375–2390. 10.1249/MSS.0000000000002116 31626055 PMC8576825

[B38] Campbell K. L. Zadravec K. Bland K. A. Chesley E. Wolf F. Janelsins M. C. (2020). The Effect of Exercise on Cancer-Related Cognitive Impairment and Applications for Physical Therapy: Systematic Review of Randomized Controlled Trials. Phys. Ther. 100 (3), 523–542. 10.1093/ptj/pzz090 32065236 PMC8559683

[B39] Castellon S. A. Ganz P. A. Bower J. E. Petersen L. Abraham L. Greendale G. A. (2004). Neurocognitive Performance in Breast Cancer Survivors Exposed to Adjuvant Chemotherapy and Tamoxifen. J. Clin. Exp. Neuropsychol. 26 (7), 955–969. 10.1080/13803390490510905 15742545

[B40] Chang J. Couture F. A. Young S. D. Lau C. Y. Lee McWatters K. (2004). Weekly Administration of Epoetin Alfa Improves Cognition and Quality of Life in Patients with Breast Cancer Receiving Chemotherapy. Support. Cancer Ther. 2 (1), 52–58. 10.3816/SCT.2004.n.023 18628159

[B41] Chen J. Buchanan J. B. Sparkman N. L. Godbout J. P. Freund G. G. Johnson R. W. (2008). Neuroinflammation and Disruption in Working Memory in Aged Mice after Acute Stimulation of the Peripheral Innate Immune System. Brain Behav. Immun. 22 (3), 301–311. 10.1016/j.bbi.2007.08.014 17951027 PMC2374919

[B42] Chen P. Chen F. Zhou B. (2021). Pharmacological Neurorescue in a Paclitaxel-Induced Chemobrain Model. Front. Behav. Neurosci. 15, 736003. 10.3389/fnbeh.2021.736003 34621160 PMC8490656

[B43] Cheng H. Li W. Gan C. Zhang B. Jia Q. Wang K. (2016). The COMT (Rs165599) Gene Polymorphism Contributes to Chemotherapy-Induced Cognitive Impairment in Breast Cancer Patients. Am. J. Transl Res. 8 (11), 5087–5097. 27904710 PMC5126352

[B44] Cheung Y. T. Ng T. Shwe M. Ho H. K. Foo K. M. Cham M. T. (2015). Association of Proinflammatory Cytokines and Chemotherapy-Associated Cognitive Impairment in Breast Cancer Patients: a Multi-Centered, Prospective, Cohort Study. Ann. Oncol. 26 (7), 1446–1451. 10.1093/annonc/mdv206 25922060 PMC4478978

[B45] Chovanec M. Vasilkova L. Setteyova L. Obertova J. Palacka P. Rejlekova K. (2018). Long‐Term Cognitive Functioning in Testicular Germ‐Cell Tumor Survivors. Oncol. 23 (5), 617–623. 10.1634/theoncologist.2017-0457 PMC5947452 29352051

[B46] Chung P. Warde P. (2011). Testicular Cancer: Seminoma. BMJ Clin. Evid. 2011. PMC3217763 21477387

[B47] Ciernikova S. Mego M. Chovanec M. (2021). Exploring the Potential Role of the Gut Microbiome in Chemotherapy-Induced Neurocognitive Disorders and Cardiovascular Toxicity. Cancers 13 (4), 782. 10.3390/cancers13040782 33668518 PMC7918783

[B48] Cimprich B. Reuter-Lorenz P. Nelson J. Clark P. M. Therrien B. Normolle D. (2010). Prechemotherapy Alterations in Brain Function in Women with Breast Cancer. J. Clin. Exp. Neuropsychol. 32 (3), 324–331. 10.1080/13803390903032537 19642048

[B49] Cimprich B. Ronis D. L. (2003). An Environmental Intervention to Restore Attention in Women with Newly Diagnosed Breast Cancer. Cancer Nurs. 26 (4), 284–292. 10.1097/00002820-200308000-00005 12886119

[B50] Collins B. MacKenzie J. Tasca G. A. Scherling C. Smith A. (2013). Cognitive Effects of Chemotherapy in Breast Cancer Patients: a Dose-Response Study. Psycho-Oncology 22 (7), 1517–1527. 10.1002/pon.3163 22936651

[B51] Correa D. D. Root J. C. Baser R. Moore D. Peck K. K. Lis E. (2013). A Prospective Evaluation of Changes in Brain Structure and Cognitive Functions in Adult Stem Cell Transplant Recipients. Brain Imaging Behav. 7 (4), 478–490. 10.1007/s11682-013-9221-8 23329358 PMC5536351

[B52] Correa D. D. Root J. C. Kryza-Lacombe M. Mehta M. Karimi S. Hensley M. L. (2017). Brain Structure and Function in Patients with Ovarian Cancer Treated with First-Line Chemotherapy: a Pilot Study. Brain Imaging Behav. 11 (6), 1652–1663. 10.1007/s11682-016-9608-4 27766586 PMC5425316

[B53] Cruzado J. A. López-Santiago S. Martínez-Marín V. José-Moreno G. Custodio A. B. Feliu J. (2014). Longitudinal Study of Cognitive Dysfunctions Induced by Adjuvant Chemotherapy in colon Cancer Patients. Support Care Cancer 22 (7), 1815–1823. 10.1007/s00520-014-2147-x 24535240

[B54] Cuzzubbo S. Javeri F. Tissier M. Roumi A. Barlog C. Doridam J. (2017). Neurological Adverse Events Associated with Immune Checkpoint Inhibitors: Review of the Literature. Eur. J. Cancer 73, 1–8. 10.1016/j.ejca.2016.12.001 28064139

[B55] Damholdt M. Mehlsen M. O'Toole M. Andreasen R. Pedersen A. Zachariae R. (2016). Web‐based Cognitive Training for Breast Cancer Survivors with Cognitive Complaints-A Randomized Controlled Trial. Psycho‐Oncology 25 (11), 1293–1300. 10.1002/pon.4058 26763774 PMC5111748

[B56] Danhauer S. C. Legault C. Bandos H. Kidwell K. Costantino J. Vaughan L. (2013). Positive and Negative Affect, Depression, and Cognitive Processes in the Cognition in the Study of Tamoxifen and Raloxifene (Co-STAR) Trial. Aging Neuropsychol. Cogn. 20 (5), 532–552. 10.1080/13825585.2012.747671 PMC3815441 23237718

[B57] De Rosa N. Della Corte L. Giannattasio A. Giampaolino P. Di Carlo C. Bifulco G. (2021). Cancer-related Cognitive Impairment (CRCI), Depression and Quality of Life in Gynecological Cancer Patients: a Prospective Study. Arch. Gynecol. Obstet. 303 (6), 1581–1588. 10.1007/s00404-020-05896-6 33404703

[B58] Debess J. Riis J. Ø. Engebjerg M. C. Ewertz M. (2010). Cognitive Function after Adjuvant Treatment for Early Breast Cancer: a Population-Based Longitudinal Study. Breast Cancer Res. Treat. 121 (1), 91–100. 10.1007/s10549-010-0756-8 20306129

[B59] Denlinger C. S. Ligibel J. A. Are M. Baker K. S. Demark-Wahnefried W. Friedman D. L. (2014). Survivorship: Cognitive Function, Version 1.2014. J. Natl. Compr. Canc Netw. 12 (7), 976–986. 10.6004/jnccn.2014.0094 24994918 PMC4465252

[B60] Deprez S. Amant F. Smeets A. Peeters R. Leemans A. Van Hecke W. (2012). Longitudinal Assessment of Chemotherapy-Induced Structural Changes in Cerebral white Matter and its Correlation with Impaired Cognitive Functioning. Jco 30 (3), 274–281. 10.1200/JCO.2011.36.8571 22184379

[B61] Dhillon H. M. Tannock I. F. Pond G. R. Renton C. Rourke S. B. Vardy J. L. (2018). Perceived Cognitive Impairment in People with Colorectal Cancer Who Do and Do Not Receive Chemotherapy. J. Cancer Surviv 12 (2), 178–185. 10.1007/s11764-017-0656-6 29080061

[B62] Diehl S. Rincón M. (2002). The Two Faces of IL-6 on Th1/Th2 Differentiation. Mol. Immunol. 39 (9), 531–536. 10.1016/s0161-5890(02)00210-9 12431386

[B63] Du J. Zhang A. Li J. Liu X. Wu S. Wang B. (2021). Doxorubicin-Induced Cognitive Impairment: The Mechanistic Insights. Front. Oncol. 11, 673340. 10.3389/fonc.2021.673340 34055643 PMC8158153

[B64] Eide S. Feng Z.-P. (2020). Doxorubicin Chemotherapy-Induced "Chemo-Brain": Meta-Analysis. Eur. J. Pharmacol. 881, 173078. 10.1016/j.ejphar.2020.173078 32505665

[B65] EORTC QLQ (2001). EORTC QLQ-C30 Scoring Manual [Online]. Brussels. Available: https://www.eortc.org/app/uploads/sites/2/2018/02/SCmanual.pdf ([Accessed].

[B66] Ercoli L. M. Petersen L. Hunter A. M. Castellon S. A. Kwan L. Kahn-Mills B. A. (2015). Cognitive Rehabilitation Group Intervention for Breast Cancer Survivors: Results of a Randomized Clinical Trial. Psycho-Oncology 24 (11), 1360–1367. 10.1002/pon.3769 25759235

[B67] Erickson K. I. Prakash R. S. Voss M. W. Chaddock L. Heo S. McLaren M. (2010). Brain-derived Neurotrophic Factor Is Associated with Age-Related Decline in Hippocampal Volume. J. Neurosci. 30 (15), 5368–5375. 10.1523/JNEUROSCI.6251-09.2010 20392958 PMC3069644

[B68] Erny D. Hrabě de Angelis A. L. Jaitin D. Wieghofer P. Staszewski O. David E. (2015). Host Microbiota Constantly Control Maturation and Function of Microglia in the CNS. Nat. Neurosci. 18 (7), 965–977. 10.1038/nn.4030 26030851 PMC5528863

[B69] Escalante C. P. Meyers C. Reuben J. M. Wang X. Qiao W. Manzullo E. (2014). A Randomized, Double-Blind, 2-period, Placebo-Controlled Crossover Trial of a Sustained-Release Methylphenidate in the Treatment of Fatigue in Cancer Patients. Cancer J. 20 (1), 8–14. 10.1097/PPO.0000000000000018 24445757 PMC4510946

[B70] FACT-Cog (2016). FACT-cog (Version 3) [Online]. FACIT Group. Available: https://www.facit.org/measures/FACT-Cog ([Accessed].

[B71] Ferguson R. J. Sigmon S. T. Pritchard A. J. LaBrie S. L. Goetze R. E. Fink C. M. (2016). A Randomized Trial of Videoconference-Delivered Cognitive Behavioral Therapy for Survivors of Breast Cancer with Self-Reported Cognitive Dysfunction. Cancer 122 (11), 1782–1791. 10.1002/cncr.29891 27135464

[B72] Fernandes H. A. Richard N. M. Edelstein K. (2019). Cognitive Rehabilitation for Cancer-Related Cognitive Dysfunction: a Systematic Review. Support Care Cancer 27 (9), 3253–3279. 10.1007/s00520-019-04866-2 31147780

[B73] Freeman L. W. White R. Ratcliff C. G. Sutton S. Stewart M. Palmer J. L. (2015). A Randomized Trial Comparing Live and Telemedicine Deliveries of an Imagery-Based Behavioral Intervention for Breast Cancer Survivors: Reducing Symptoms and Barriers to Care. Psycho-Oncology 24 (8), 910–918. 10.1002/pon.3656 25146413 PMC4379121

[B74] Ganz P. A. Bower J. E. Kwan L. Castellon S. A. Silverman D. H. S. Geist C. (2013a). Does Tumor Necrosis Factor-Alpha (TNF-α) Play a Role in post-chemotherapy Cerebral Dysfunction? Brain Behav. Immun. 30 (Suppl. l), S99–S108. 10.1016/j.bbi.2012.07.015 22884417 PMC3522786

[B75] Ganz P. A. Kwan L. Castellon S. A. Oppenheim A. Bower J. E. Silverman D. H. S. (2013b). Cognitive Complaints after Breast Cancer Treatments: Examining the Relationship with Neuropsychological Test Performance. J. Natl. Cancer Inst. 105 (11), 791–801. 10.1093/jnci/djt073 23606729 PMC3672076

[B76] Ganz P. A. Petersen L. Castellon S. A. Bower J. E. Silverman D. H. S. Cole S. W. (2014). Cognitive Function after the Initiation of Adjuvant Endocrine Therapy in Early-Stage Breast Cancer: an Observational Cohort Study. Jco 32 (31), 3559–3567. 10.1200/JCO.2014.56.1662 PMC4209106 25267747

[B77] Geraghty A. C. Gibson E. M. Ghanem R. A. Greene J. J. Ocampo A. Goldstein A. K. (2019). Loss of Adaptive Myelination Contributes to Methotrexate Chemotherapy-Related Cognitive Impairment. Neuron 103 (2), 250–265. 10.1016/j.neuron.2019.04.032 31122677 PMC6697075

[B78] Gibson E. M. Nagaraja S. Ocampo A. Tam L. T. Wood L. S. Pallegar P. N. (2019). Methotrexate Chemotherapy Induces Persistent Tri-glial Dysregulation that Underlies Chemotherapy-Related Cognitive Impairment. Cell 176 (1-2), 43–55. 10.1016/j.cell.2018.10.049 30528430 PMC6329664

[B79] Gilligan T. Lin D. W. Aggarwal R. Chism D. Cost N. Derweesh I. H. (2019). Testicular Cancer, Version 2.2020, NCCN Clinical Practice Guidelines in Oncology. J. Natl. Compr. Canc Netw. 17 (12), 1529–1554. 10.6004/jnccn.2019.0058 31805523

[B80] Gonzalez B. D. Jim H. S. L. Booth-Jones M. Small B. J. Sutton S. K. Lin H.-Y. (2015). Course and Predictors of Cognitive Function in Patients with Prostate Cancer Receiving Androgen-Deprivation Therapy: A Controlled Comparison. Jco 33 (18), 2021–2027. 10.1200/JCO.2014.60.1963 PMC4461804 25964245

[B81] Grasso M. Piscopo P. Confaloni A. Denti M. (2014). Circulating miRNAs as Biomarkers for Neurodegenerative Disorders. Molecules 19 (5), 6891–6910. 10.3390/molecules19056891 24858274 PMC6271879

[B82] Gunlusoy B. Ceylan Y. Koskderelioglu A. Gedizlioglu M. Degirmenci T. Ortan P. (2017). Cognitive Effects of Androgen Deprivation Therapy in Men with Advanced Prostate Cancer. Urology 103, 167–172. 10.1016/j.urology.2016.12.060 28188757

[B83] Han L. Lam E. W.-F. Sun Y. (2019). Extracellular Vesicles in the Tumor Microenvironment: Old Stories, but New Tales. Mol. Cancer 18 (1), 59. 10.1186/s12943-019-0980-8 30925927 PMC6441234

[B84] Hasegawa S. Goto S. Tsuji H. Okuno T. Asahara T. Nomoto K. (2015). Intestinal Dysbiosis and Lowered Serum Lipopolysaccharide-Binding Protein in Parkinson's Disease. PLoS One 10 (11), e0142164. 10.1371/journal.pone.0142164 26539989 PMC4634857

[B85] Henneghan A. Haley A. P. Kesler S. (2020). Exploring Relationships Among Peripheral Amyloid Beta, Tau, Cytokines, Cognitive Function, and Psychosomatic Symptoms in Breast Cancer Survivors. Biol. Res. Nurs. 22 (1), 126–138. 10.1177/1099800419887230 31707784 PMC7068749

[B86] Hermelink K. Untch M. Lux M. P. Kreienberg R. Beck T. Bauerfeind I. (2007). Cognitive Function during Neoadjuvant Chemotherapy for Breast Cancer. Cancer 109 (9), 1905–1913. 10.1002/cncr.22610 17351951

[B87] Hermelink K. Voigt V. Kaste J. Neufeld F. Wuerstlein R. Buhner M. (2015). Elucidating Pretreatment Cognitive Impairment in Breast Cancer Patients: the Impact of Cancer-Related post-traumatic Stress. JNCI J. Natl. Cancer Inst. 107 (7), djv099. 10.1093/jnci/djv099 25882713

[B88] Hess L. M. Huang H. Q. Hanlon A. L. Robinson W. R. Johnson R. Chambers S. K. (2015). Cognitive Function during and Six Months Following Chemotherapy for Front-Line Treatment of Ovarian, Primary Peritoneal or Fallopian Tube Cancer: An NRG Oncology/gynecologic Oncology Group Study. Gynecol. Oncol. 139 (3), 541–545. 10.1016/j.ygyno.2015.10.003 26456812 PMC4698796

[B89] Hodgson K. D. Hutchinson A. D. Wilson C. J. Nettelbeck T. (2013). A Meta-Analysis of the Effects of Chemotherapy on Cognition in Patients with Cancer. Cancer Treat. Rev. 39 (3), 297–304. 10.1016/j.ctrv.2012.11.001 23219452

[B90] Hoffman C. J. Ersser S. J. Hopkinson J. B. Nicholls P. G. Harrington J. E. Thomas P. W. (2012). Effectiveness of Mindfulness-Based Stress Reduction in Mood, Breast- and Endocrine-Related Quality of Life, and Well-Being in Stage 0 to III Breast Cancer: a Randomized, Controlled Trial. Jco 30 (12), 1335–1342. 10.1200/JCO.2010.34.0331 22430268

[B91] Holtfrerich S. K. C. Knipper S. Purwins J. Castens J. Beyer B. Schlomm T. (2020). The Impact of Long‐term Androgen Deprivation Therapy on Cognitive Function and Socioeconomic Decision Making in Prostate Cancer Patients. Psycho‐Oncology 29 (8), 1338–1346. 10.1002/pon.5442 32539186

[B92] Horowitz T. S. Suls J. Treviño M. (2018). A Call for a Neuroscience Approach to Cancer-Related Cognitive Impairment. Trends Neurosciences 41 (8), 493–496. 10.1016/j.tins.2018.05.001 29907436

[B93] Hsu T. Ennis M. Hood N. Graham M. Goodwin P. J. (2013). Quality of Life in Long-Term Breast Cancer Survivors. Jco 31 (28), 3540–3548. 10.1200/JCO.2012.48.1903 23980087

[B94] Hurria A. Patel S. K. Mortimer J. Luu T. Somlo G. Katheria V. (2014). The Effect of Aromatase Inhibition on the Cognitive Function of Older Patients with Breast Cancer. Clin. Breast Cancer 14 (2), 132–140. 10.1016/j.clbc.2013.10.010 24291380 PMC4103787

[B95] Hutchinson A. D. Hosking J. R. Kichenadasse G. Mattiske J. K. Wilson C. (2012). Objective and Subjective Cognitive Impairment Following Chemotherapy for Cancer: a Systematic Review. Cancer Treat. Rev. 38 (7), 926–934. 10.1016/j.ctrv.2012.05.002 22658913

[B96] Ibrahim E. Y. Domenicano I. Nyhan K. Elfil M. Mougalian S. S. Cartmel B. (2021). Cognitive Effects and Depression Associated with Taxane-Based Chemotherapy in Breast Cancer Survivors: A Meta-Analysis. Front. Oncol. 11, 642382. 10.3389/fonc.2021.642382 33996556 PMC8121254

[B97] Ishikawa T. Kokura S. Sakamoto N. Okajima M. Matsuyama T. Sakai H. (2012). Relationship between Circulating Cytokine Levels and Physical or Psychological Functioning in Patients with Advanced Cancer. Clin. Biochem. 45 (3), 207–211. 10.1016/j.clinbiochem.2011.09.007 21963382

[B98] Jacobs W. Das E. Schagen S. B. (2017). Increased Cognitive Problem Reporting after Information about Chemotherapy-Induced Cognitive Decline: The Moderating Role of Stigma Consciousness. Psychol. Health 32 (1), 78–93. 10.1080/08870446.2016.1244535 27701901

[B99] Janelsins M. C. Heckler C. E. Peppone L. J. Kamen C. Mustian K. M. Mohile S. G. (2017). Cognitive Complaints in Survivors of Breast Cancer after Chemotherapy Compared with Age-Matched Controls: An Analysis from a Nationwide, Multicenter, Prospective Longitudinal Study. Jco 35 (5), 506–514. 10.1200/JCO.2016.68.5826 PMC5455314 28029304

[B100] Janelsins M. C. Kohli S. Mohile S. G. Usuki K. Ahles T. A. Morrow G. R. (2011). An Update on Cancer- and Chemotherapy-Related Cognitive Dysfunction: Current Status. Semin. Oncol. 38 (3), 431–438. 10.1053/j.seminoncol.2011.03.014 21600374 PMC3120018

[B101] Janelsins M. C. Mustian K. M. Palesh O. G. Mohile S. G. Peppone L. J. Sprod L. K. (2012). Differential Expression of Cytokines in Breast Cancer Patients Receiving Different Chemotherapies: Implications for Cognitive Impairment Research. Support Care Cancer 20 (4), 831–839. 10.1007/s00520-011-1158-0 21533812 PMC3218259

[B102] Jansen C. E. Cooper B. A. Dodd M. J. Miaskowski C. A. (2011). A Prospective Longitudinal Study of Chemotherapy-Induced Cognitive Changes in Breast Cancer Patients. Support Care Cancer 19 (10), 1647–1656. 10.1007/s00520-010-0997-4 20820813

[B103] Jayadevappa R. Chhatre S. Malkowicz S. B. Parikh R. B. Guzzo T. Wein A. J. (2019). Association between Androgen Deprivation Therapy Use and Diagnosis of Dementia in Men with Prostate Cancer. JAMA Netw. Open 2 (7), e196562. 10.1001/jamanetworkopen.2019.6562 31268539 PMC6613289

[B104] Jim H. S. L. Phillips K. M. Chait S. Faul L. A. Popa M. A. Lee Y.-H. (2012). Meta-analysis of Cognitive Functioning in Breast Cancer Survivors Previously Treated with Standard-Dose Chemotherapy. Jco 30 (29), 3578–3587. 10.1200/JCO.2011.39.5640 PMC3462044 22927526

[B105] Johns S. A. Brown L. F. Beck-Coon K. Talib T. L. Monahan P. O. Giesler R. B. (2016). Randomized Controlled Pilot Trial of Mindfulness-Based Stress Reduction Compared to Psychoeducational Support for Persistently Fatigued Breast and Colorectal Cancer Survivors. Support Care Cancer 24 (10), 4085–4096. 10.1007/s00520-016-3220-4 27189614 PMC5221754

[B106] Joly F. Castel H. Tron L. Lange M. Vardy J. (2020). Potential Effect of Immunotherapy Agents on Cognitive Function in Cancer Patients. J. Natl. Cancer Inst. 112 (2), 123–127. 10.1093/jnci/djz168 31504664 PMC7019093

[B107] Joly F. Giffard B. Rigal O. De Ruiter M. B. Small B. J. Dubois M. (2015). Impact of Cancer and its Treatments on Cognitive Function: Advances in Research from the Paris International Cognition and Cancer Task Force Symposium and Update since 2012. J. Pain Symptom Manage. 50 (6), 830–841. 10.1016/j.jpainsymman.2015.06.019 26344551

[B108] Joly F. Heutte N. Duclos B. Noal S. Léger-Hardy I. Dauchy S. (2016). Prospective Evaluation of the Impact of Antiangiogenic Treatment on Cognitive Functions in Metastatic Renal Cancer. Eur. Urol. Focus 2 (6), 642–649. 10.1016/j.euf.2016.04.009 28723499

[B109] Karschnia P. Parsons M. W. Dietrich J. (2019). Pharmacologic Management of Cognitive Impairment Induced by Cancer Therapy. Lancet Oncol. 20 (2), e92–e102. 10.1016/S1470-2045(18)30938-0 30723041

[B110] Kenny A. McArdle H. Calero M. Rabano A. Madden S. Adamson K. (2019). Elevated Plasma microRNA-206 Levels Predict Cognitive Decline and Progression to Dementia from Mild Cognitive Impairment. Biomolecules 9 (11), 734. 10.3390/biom9110734 PMC6920950 31766231

[B111] Kesler S. Hadi Hosseini S. M. Heckler C. Janelsins M. Palesh O. Mustian K. (2013a). Cognitive Training for Improving Executive Function in Chemotherapy-Treated Breast Cancer Survivors. Clin. Breast Cancer 13 (4), 299–306. 10.1016/j.clbc.2013.02.004 23647804 PMC3726272

[B112] Kesler S. Janelsins M. Koovakkattu D. Palesh O. Mustian K. Morrow G. (2013b). Reduced Hippocampal Volume and Verbal Memory Performance Associated with Interleukin-6 and Tumor Necrosis Factor-Alpha Levels in Chemotherapy-Treated Breast Cancer Survivors. Brain Behav. Immun. 30 (Suppl. l), S109–S116. 10.1016/j.bbi.2012.05.017 22698992 PMC3665606

[B113] Koh Y. Q. Tan C. J. Toh Y. L. Sze S. K. Ho H. K. Limoli C. L. (2020). Role of Exosomes in Cancer-Related Cognitive Impairment. Ijms 21 (8), 2755. 10.3390/ijms21082755 PMC7215650 32326653

[B114] Kohli S. Fisher S. G. Tra Y. Adams M. J. Mapstone M. E. Wesnes K. A. (2009). The Effect of Modafinil on Cognitive Function in Breast Cancer Survivors. Cancer 115 (12), 2605–2616. 10.1002/cncr.24287 19309747 PMC2796482

[B115] Koppelmans V. Breteler M. M. B. Boogerd W. Seynaeve C. Gundy C. Schagen S. B. (2012a). Neuropsychological Performance in Survivors of Breast Cancer More Than 20 Years after Adjuvant Chemotherapy. Jco 30 (10), 1080–1086. 10.1200/JCO.2011.37.0189 22370315

[B116] Koppelmans V. de Ruiter M. B. van der Lijn F. Boogerd W. Seynaeve C. van der Lugt A. (2012b). Global and Focal Brain Volume in Long-Term Breast Cancer Survivors Exposed to Adjuvant Chemotherapy. Breast Cancer Res. Treat. 132 (3), 1099–1106. 10.1007/s10549-011-1888-1 22205140

[B117] Kovalchuk A. Rodriguez-Juarez R. Ilnytskyy Y. Byeon B. Shpyleva S. Melnyk S. (2016). Sex-specific Effects of Cytotoxic Chemotherapy Agents Cyclophospha-Mide and Mitomycin C on Gene Expression, Oxidative DNA Damage, and Epigenetic Alterations in the Prefrontal Cortex and hippocampus - an Aging Connection. Aging 8 (4), 697–708. 10.18632/aging.100920 27032448 PMC4925823

[B118] Krull K. R. Hockenberry M. J. Miketova P. Carey M. Moore I. M. (2013). Chemotherapy-related Changes in central Nervous System Phospholipids and Neurocognitive Function in Childhood Acute Lymphoblastic Leukemia. Leuk. Lymphoma 54 (3), 535–540. 10.3109/10428194.2012.717080 22856670 PMC3845091

[B119] Lange M. Giffard B. Noal S. Rigal O. Kurtz J.-E. Heutte N. (2014). Baseline Cognitive Functions Among Elderly Patients with Localised Breast Cancer. Eur. J. Cancer 50 (13), 2181–2189. 10.1016/j.ejca.2014.05.026 24958735

[B120] Lange M. Heutte N. Noal S. Rigal O. Kurtz J. E. Lévy C. (2019a). Cognitive Changes after Adjuvant Treatment in Older Adults with Early‐Stage Breast Cancer. Oncol. 24 (1), 62–68. 10.1634/theoncologist.2017-0570 PMC6324624 29934409

[B121] Lange M. Heutte N. Rigal O. Noal S. Kurtz J. E. Lévy C. (2016). Decline in Cognitive Function in Older Adults with Early‐Stage Breast Cancer after Adjuvant Treatment. The Oncologist 21 (11), 1337–1348. 10.1634/theoncologist.2016-0014 27473044 PMC5189619

[B122] Lange M. Licaj I. Clarisse B. Humbert X. Grellard J. M. Tron L. (2019b). Cognitive Complaints in Cancer Survivors and Expectations for Support: Results from a Web-Based Survey. Cancer Med. 8 (5), 2654–2663. 10.1002/cam4.2069 30884207 PMC6536919

[B123] Lawrence J. A. Griffin L. Balcueva E. P. Groteluschen D. L. Samuel T. A. Lesser G. J. (2016). A Study of Donepezil in Female Breast Cancer Survivors with Self-Reported Cognitive Dysfunction 1 to 5 Years Following Adjuvant Chemotherapy. J. Cancer Surviv 10 (1), 176–184. 10.1007/s11764-015-0463-x 26130292 PMC4930878

[B124] Le Rhun E. Delbeuck X. Lefeuvre-Plesse C. Kramar A. Skrobala E. Pasquier F. (2015). A Phase III Randomized Multicenter Trial Evaluating Cognition in post-menopausal Breast Cancer Patients Receiving Adjuvant Hormonotherapy. Breast Cancer Res. Treat. 152 (3), 569–580. 10.1007/s10549-015-3493-1 26160250

[B125] Lee B. E. Choi B. Y. Hong D. K. Kim J. H. Lee S. H. Kho A. R. (2017). The Cancer Chemotherapeutic Agent Paclitaxel (Taxol) Reduces Hippocampal Neurogenesis via Down-Regulation of Vesicular Zinc. Sci. Rep. 7 (1), 11667. 10.1038/s41598-017-12054-7 28916767 PMC5601929

[B126] Li D.-Q. Dai Z. Y. Zhu B. Y. Zhen J. P. Yang W. F. Li D. Q. (2014). Effects of Sertraline on Executive Function and Quality of Life in Patients with Advanced Cancer. Med. Sci. Monit. 20, 1267–1273. 10.12659/MSM.890575 25047152 PMC4114699

[B127] Li M. Caeyenberghs K. (2018). Longitudinal Assessment of Chemotherapy-Induced Changes in Brain and Cognitive Functioning: A Systematic Review. Neurosci. Biobehavioral Rev. 92, 304–317. 10.1016/j.neubiorev.2018.05.019 29791867

[B128] Lindner O. C. Phillips B. McCabe M. G. Mayes A. Wearden A. Varese F. (2014). A Meta-Analysis of Cognitive Impairment Following Adult Cancer Chemotherapy. Neuropsychology 28 (5), 726–740. 10.1037/neu0000064 24635712 PMC4143183

[B129] Lomeli N. Di K. Czerniawski J. Guzowski J. F. Bota D. A. (2017). Cisplatin-induced Mitochondrial Dysfunction Is Associated with Impaired Cognitive Function in Rats. Free Radic. Biol. Med. 102, 274–286. 10.1016/j.freeradbiomed.2016.11.046 27908784 PMC5308450

[B130] López Zunini R. A. Scherling C. Wallis N. Collins B. MacKenzie J. Bielajew C. (2013). Differences in Verbal Memory Retrieval in Breast Cancer Chemotherapy Patients Compared to Healthy Controls: a Prospective fMRI Study. Brain Imaging Behav. 7 (4), 460–477. 10.1007/s11682-012-9213-0 23242968

[B131] Lower E. E. Fleishman S. Cooper A. Zeldis J. Faleck H. Yu Z. (2009). Efficacy of Dexmethylphenidate for the Treatment of Fatigue after Cancer Chemotherapy: a Randomized Clinical Trial. J. Pain Symptom Manage. 38 (5), 650–662. 10.1016/j.jpainsymman.2009.03.011 19896571

[B132] Lundorff L. Jønsson B. Sjøgren P. (2009). Modafinil for Attentional and Psychomotor Dysfunction in Advanced Cancer: a Double-Blind, Randomised, Cross-Over Trial. Palliat. Med. 23 (8), 731–738. 10.1177/0269216309106872 19648224

[B133] Lyon D. E. Cohen R. Chen H. Kelly D. L. McCain N. L. Starkweather A. (2016). Relationship of Systemic Cytokine Concentrations to Cognitive Function over Two Years in Women with Early Stage Breast Cancer. J. Neuroimmunology 301, 74–82. 10.1016/j.jneuroim.2016.11.002 27890459 PMC5181109

[B134] Makale M. T. McDonald C. R. Hattangadi-Gluth J. A. Kesari S. (2017). Mechanisms of Radiotherapy-Associated Cognitive Disability in Patients with Brain Tumours. Nat. Rev. Neurol. 13 (1), 52–64. 10.1038/nrneurol.2016.185 27982041 PMC5805381

[B135] Mandelblatt J. S. Stern R. A. Luta G. McGuckin M. Clapp J. D. Hurria A. (2014). Cognitive Impairment in Older Patients with Breast Cancer before Systemic Therapy: Is There an Interaction between Cancer and Comorbidity? Jco 32 (18), 1909–1918. 10.1200/JCO.2013.54.2050 PMC4050204 24841981

[B136] Mar Fan H. G. Clemons M. Xu W. Chemerynsky I. Breunis H. Braganza S. (2008). A Randomised, Placebo-Controlled, Double-Blind Trial of the Effects of D-Methylphenidate on Fatigue and Cognitive Dysfunction in Women Undergoing Adjuvant Chemotherapy for Breast Cancer. Support Care Cancer 16 (6), 577–583. 10.1007/s00520-007-0341-9 17972110

[B137] Marzouk S. Naglie G. Tomlinson G. Duff Canning S. Breunis H. Timilshina N. (2018). Impact of Androgen Deprivation Therapy on Self-Reported Cognitive Function in Men with Prostate Cancer. J. Urol. 200 (2), 327–334. 10.1016/j.juro.2018.02.073 29477720

[B138] Matsos A. Johnston I. N. (2019). Chemotherapy-induced Cognitive Impairments: A Systematic Review of the Animal Literature. Neurosci. Biobehavioral Rev. 102, 382–399. 10.1016/j.neubiorev.2019.05.001 31063740

[B139] McDonald B. C. Conroy S. K. Ahles T. A. West J. D. Saykin A. J. (2012). Alterations in Brain Activation during Working Memory Processing Associated with Breast Cancer and Treatment: a Prospective Functional Magnetic Resonance Imaging Study. Jco 30 (20), 2500–2508. 10.1200/JCO.2011.38.5674 PMC3397784 22665542

[B140] McDonald B. C. Conroy S. K. Smith D. J. West J. D. Saykin A. J. (2013). Frontal gray Matter Reduction after Breast Cancer Chemotherapy and Association with Executive Symptoms: a Replication and Extension Study. Brain Behav. Immun. 30 (Suppl. l), S117–S125. 10.1016/j.bbi.2012.05.007 22613170 PMC3629547

[B141] McDuff S. G. R. Taich Z. J. Lawson J. D. Sanghvi P. Wong E. T. Barker F. G. 2nd (2013). Neurocognitive Assessment Following Whole Brain Radiation Therapy and Radiosurgery for Patients with Cerebral Metastases: Table 1. J. Neurol. Neurosurg. Psychiatry 84 (12), 1384–1391. 10.1136/jnnp-2013-305166 23715918

[B142] McGinnis G. J. Friedman D. Young K. H. Torres E. R. S. Thomas C. R. Jr. Gough M. J. (2017). Neuroinflammatory and Cognitive Consequences of Combined Radiation and Immunotherapy in a Novel Preclinical Model. Oncotarget 8 (6), 9155–9173. 10.18632/oncotarget.13551 27893434 PMC5354722

[B143] McGinty H. L. Phillips K. M. Jim H. S. L. Cessna J. M. Asvat Y. Cases M. G. (2014). Cognitive Functioning in Men Receiving Androgen Deprivation Therapy for Prostate Cancer: a Systematic Review and Meta-Analysis. Support Care Cancer 22 (8), 2271–2280. 10.1007/s00520-014-2285-1 24859915 PMC4090762

[B144] Mehnert A. Scherwath A. Schirmer L. Schleimer B. Petersen C. Schulz-Kindermann F. (2007). The Association between Neuropsychological Impairment, Self-Perceived Cognitive Deficits, Fatigue and Health Related Quality of Life in Breast Cancer Survivors Following Standard Adjuvant versus High-Dose Chemotherapy. Patient Education Couns. 66 (1), 108–118. 10.1016/j.pec.2006.11.005 17320337

[B145] Meneses K. Benz R. Bail J. R. Vo J. B. Triebel K. Fazeli P. (2018). Speed of Processing Training in Middle-Aged and Older Breast Cancer Survivors (SOAR): Results of a Randomized Controlled Pilot. Breast Cancer Res. Treat. 168 (1), 259–267. 10.1007/s10549-017-4564-2 29128897 PMC5823754

[B146] Menning S. de Ruiter M. B. Veltman D. J. Koppelmans V. Kirschbaum C. Boogerd W. (2015). Multimodal MRI and Cognitive Function in Patients with Breast Cancer Prior to Adjuvant Treatment - the Role of Fatigue. NeuroImage: Clin. 7, 547–554. 10.1016/j.nicl.2015.02.005 25844311 PMC4375788

[B147] Merriman J. D. Aouizerat B. E. Cataldo J. K. Dunn L. Cooper B. A. West C. (2014). Association between an Interleukin 1 Receptor, Type I Promoter Polymorphism and Self-Reported Attentional Function in Women with Breast Cancer. Cytokine 65 (2), 192–201. 10.1016/j.cyto.2013.11.003 24315345 PMC3927406

[B148] Merriman J. D. Von Ah D. Miaskowski C. Aouizerat B. E. (2013). Proposed Mechanisms for Cancer- and Treatment-Related Cognitive Changes. Semin. Oncol. Nurs. 29 (4), 260–269. 10.1016/j.soncn.2013.08.006 24183157 PMC3817493

[B149] Meyers C. A. Albitar M. Estey E. (2005). Cognitive Impairment, Fatigue, and Cytokine Levels in Patients with Acute Myelogenous Leukemia or Myelodysplastic Syndrome. Cancer 104 (4), 788–793. 10.1002/cncr.21234 15973668

[B150] Milbury K. Chaoul A. Biegler K. Wangyal T. Spelman A. Meyers C. A. (2013). Tibetan Sound Meditation for Cognitive Dysfunction: Results of a Randomized Controlled Pilot Trial. Psycho-Oncology 22 (10), 2354–2363. 10.1002/pon.3296 23657969 PMC6083855

[B151] Miller K. D. Nogueira L. Mariotto A. B. Rowland J. H. Yabroff K. R. Alfano C. M. (2019). Cancer Treatment and Survivorship Statistics, 2019. CA A. Cancer J. Clin. 69 (5), 363–385. 10.3322/caac.21565 31184787

[B152] Monje M. Dietrich J. (2012). Cognitive Side Effects of Cancer Therapy Demonstrate a Functional Role for Adult Neurogenesis. Behav. Brain Res. 227 (2), 376–379. 10.1016/j.bbr.2011.05.012 21621557 PMC3221863

[B153] Moore I. M. Miketova P. Hockenberry M. Krull K. Pasvogel A. Carey M. (2008). Methotrexate-induced Alterations in Beta-Oxidation Correlate with Cognitive Abilities in Children with Acute Lymphoblastic Leukemia. Biol. Res. Nurs. 9 (4), 311–319. 10.1177/1099800407313268 18398226

[B154] Morin R. T. Midlarsky E. (2018). Treatment with Chemotherapy and Cognitive Functioning in Older Adult Cancer Survivors. Arch. Phys. Med. Rehabil. 99 (2), 257–263. 10.1016/j.apmr.2017.06.016 28735719

[B155] Morote J. Tabernero Á. J. Álvarez Ossorio J. L. Ciria J. P. Domínguez-Escrig J. L. Vázquez F. (2017). Cognitive Function in Patients with Prostate Cancer Receiving Luteinizing Hormone-Releasing Hormone Analogues: A Prospective, Observational, Multicenter Study. Int. J. Radiat. Oncology*Biology*Physics 98 (3), 590–594. 10.1016/j.ijrobp.2017.02.219 28581399

[B156] Mulder S. F. Bertens D. Desar I. M. Vissers K. C. Mulders P. F. Punt C. J. (2014). Impairment of Cognitive Functioning during Sunitinib or Sorafenib Treatment in Cancer Patients: a Cross Sectional Study. BMC Cancer 14, 219. 10.1186/1471-2407-14-219 24661373 PMC3987809

[B157] Natori A. Ogata T. Sumitani M. Kogure T. Yamauchi T. Yamauchi H. (2015). Potential Role of pNF-H, a Biomarker of Axonal Damage in the central Nervous System, as a Predictive Marker of Chemotherapy-Induced Cognitive Impairment. Clin. Cancer Res. 21 (6), 1348–1352. 10.1158/1078-0432.CCR-14-2775 25589615

[B158] Nava Catorce M. Gevorkian G. (2016). LPS-induced Murine Neuroinflammation Model: Main Features and Suitability for Pre-clinical Assessment of Nutraceuticals. Cn 14 (2), 155–164. 10.2174/1570159x14666151204122017 PMC4825946 26639457

[B159] Nead K. T. Gaskin G. Chester C. Swisher-McClure S. Dudley J. T. Leeper N. J. (2016). Androgen Deprivation Therapy and Future Alzheimer's Disease Risk. Jco 34 (6), 566–571. 10.1200/JCO.2015.63.6266 PMC5070576 26644522

[B160] Nead K. T. Gaskin G. Chester C. Swisher-McClure S. Leeper N. J. Shah N. H. (2017). Association between Androgen Deprivation Therapy and Risk of Dementia. JAMA Oncol. 3 (1), 49–55. 10.1001/jamaoncol.2016.3662 27737437

[B161] Nelson C. J. Lee J. S. Gamboa M. C. Roth A. J. (2008). Cognitive Effects of Hormone Therapy in Men with Prostate Cancer. Cancer 113 (5), 1097–1106. 10.1002/cncr.23658 18666210 PMC4333639

[B162] Ng T. Cheung Y. T. Ng Q. S. Ho H. K. Chan A. (2014). Vascular Endothelial Growth Factor Inhibitors and Cognitive Impairment: Evidence and Controversies. Expert Opin. Drug Saf. 13 (1), 83–92. 10.1517/14740338.2013.828034 23931162

[B163] Ng T. Dorajoo S. R. Cheung Y. T. Lam Y. C. Yeo H. L. Shwe M. (2018). Distinct and Heterogeneous Trajectories of Self-Perceived Cognitive Impairment Among Asian Breast Cancer Survivors. Psycho-Oncology 27 (4), 1185–1192. 10.1002/pon.4635 29315963

[B164] Ng T. Teo S. M. Yeo H. L. Shwe M. Gan Y. X. Cheung Y. T. (2016). Brain-derived Neurotrophic Factor Genetic Polymorphism (Rs6265) Is Protective against Chemotherapy-Associated Cognitive Impairment in Patients with Early-Stage Breast Cancer. Neuro Oncol. 18 (2), 244–251. 10.1093/neuonc/nov162 26289590 PMC4724179

[B165] Nguyen L. D. Fischer T. T. Ehrlich B. E. (2021). Pharmacological rescue of Cognitive Function in a Mouse Model of Chemobrain. Mol. Neurodegeneration 16 (1), 41. 10.1186/s13024-021-00463-2 PMC8235868 34174909

[B166] Ongnok B. Chattipakorn N. Chattipakorn S. C. (2020). Doxorubicin and Cisplatin Induced Cognitive Impairment: The Possible Mechanisms and Interventions. Exp. Neurol. 324, 113118. 10.1016/j.expneurol.2019.113118 31756316

[B167] Oppegaard K. Harris C. S. Shin J. Paul S. M. Cooper B. A. Chan A. (2021). Cancer-related Cognitive Impairment Is Associated with Perturbations in Inflammatory Pathways. Cytokine 148, 155653. 10.1016/j.cyto.2021.155653 34388477 PMC10792770

[B168] Park J.-H. Jung Y. S. Kim K. S. Bae S. H. (2017). Effects of Compensatory Cognitive Training Intervention for Breast Cancer Patients Undergoing Chemotherapy: a Pilot Study. Support Care Cancer 25 (6), 1887–1896. 10.1007/s00520-017-3589-8 28132089

[B169] Pascale A. Marchesi N. Marelli C. Coppola A. Luzi L. Govoni S. (2018). Microbiota and Metabolic Diseases. Endocrine 61 (3), 357–371. 10.1007/s12020-018-1605-5 29721802

[B170] Pedersen A. D. Rossen P. Mehlsen M. Y. Pedersen C. G. Zachariae R. von der Maase H. (2009). Long-term Cognitive Function Following Chemotherapy in Patients with Testicular Cancer. J. Int. Neuropsychol. Soc. 15 (2), 296–301. 10.1017/S1355617709090316 19203434

[B171] Phillips K.-A. Regan M. M. Regan M. M. Ribi K. Francis P. A. Puglisi F. (2016). Adjuvant Ovarian Function Suppression and Cognitive Function in Women with Breast Cancer. Br. J. Cancer 114 (9), 956–964. 10.1038/bjc.2016.71 27092785 PMC4984913

[B172] Piscopo P. Lacorte E. Feligioni M. Mayer F. Crestini A. Piccolo L. (2019). MicroRNAs and Mild Cognitive Impairment: A Systematic Review. Ageing Res. Rev. 50, 131–141. 10.1016/j.arr.2018.11.005 30472218

[B173] Plata-Bello J. Plata-Bello A. Pérez-Martín Y. Fajardo V. Concepción-Massip T. (2019). Androgen Deprivation Therapy Increases Brain Ageing. Aging 11 (15), 5613–5627. 10.18632/aging.102142 31377745 PMC6710035

[B174] Protas P. T. Muszynska-Roslan K. Holownia A. Grabowska A. Wielgat P. Krawczuk-Rybak M. (2009). Negative Correlation between Cerebrospinal Fluid Tau Protein and Cognitive Functioning in Children with Acute Lymphoblastic Leukemia. Pediatr. Blood Cancer 53 (1), 105–108. 10.1002/pbc.22029 19309718

[B175] Pullens M. J. J. De Vries J. Van Warmerdam L. J. C. Van De Wal M. A. Roukema J. A. (2013). Chemotherapy and Cognitive Complaints in Women with Breast Cancer. Psycho-Oncology 22 (8), 1783–1789. 10.1002/pon.3214 23109296

[B176] Quesnel C. Savard J. Ivers H. (2009). Cognitive Impairments Associated with Breast Cancer Treatments: Results from a Longitudinal Study. Breast Cancer Res. Treat. 116 (1), 113–123. 10.1007/s10549-008-0114-2 18629633

[B177] Rajagopal C. Harikumar K. B. (2018). The Origin and Functions of Exosomes in Cancer. Front. Oncol. 8, 66. 10.3389/fonc.2018.00066 29616188 PMC5869252

[B178] Reid-Arndt S. A. Hsieh C. Perry M. C. (2010). Neuropsychological Functioning and Quality of Life during the First Year after Completing Chemotherapy for Breast Cancer. Psycho-Oncology 19 (5), 535–544. 10.1002/pon.1581 19472296 PMC2861143

[B179] Reitan R. M. (1992). Trail Making Test: Manual for Administration and Scoring. Tucson, AZ: Reitan Neuropsychology Laboratory.

[B180] Saykin A. J. de Ruiter M. B. McDonald B. C. Deprez S. Silverman D. H. S. (2013). Neuroimaging Biomarkers and Cognitive Function in Non-CNS Cancer and its Treatment: Current Status and Recommendations for Future Research. Brain Imaging Behav. 7 (4), 363–373. 10.1007/s11682-013-9283-7 24327327 PMC3909524

[B181] Schagen S. B. Boogerd W. Muller M. J. Ten Bokkel Huinink W. Moonen L. Meinhardt W. (2008). Cognitive Complaints and Cognitive Impairment Following BEP Chemotherapy in Patients with Testicular Cancer. Acta Oncologica 47 (1), 63–70. 10.1080/02841860701518058 17934892

[B182] Schagen S. B. Das E. Vermeulen I. (2012). Information about Chemotherapy-Associated Cognitive Problems Contributes to Cognitive Problems in Cancer Patients. Psycho-Oncology 21 (10), 1132–1135. 10.1002/pon.2011 21769988

[B183] Scherling C. Collins B. Mackenzie J. Bielajew C. Smith A. (2011). Pre-chemotherapy Differences in Visuospatial Working Memory in Breast Cancer Patients Compared to Controls: an FMRI Study. Front. Hum. Neurosci. 5, 122. 10.3389/fnhum.2011.00122 22053153 PMC3205481

[B184] Scherling C. Collins B. Mackenzie J. Bielajew C. Smith A. (2012). Prechemotherapy Differences in Response Inhibition in Breast Cancer Patients Compared to Controls: a Functional Magnetic Resonance Imaging Study. J. Clin. Exp. Neuropsychol. 34 (5), 543–560. 10.1080/13803395.2012.666227 22380580

[B185] Scherwath A. Schirmer L. Kruse M. Ernst G. Eder M. Dinkel A. (2013). Cognitive Functioning in Allogeneic Hematopoietic Stem Cell Transplantation Recipients and its Medical Correlates: a Prospective Multicenter Study. Psycho-Oncology 22 (7), 1509–1516. 10.1002/pon.3159 22945857

[B186] Schiepers O. J. G. Harris S. E. Gow A. J. Pattie A. Brett C. E. Starr J. M. (2012). APOE E4 Status Predicts Age-Related Cognitive Decline in the Ninth Decade: Longitudinal Follow-Up of the Lothian Birth Cohort 1921. Mol. Psychiatry 17 (3), 315–324. 10.1038/mp.2010.137 21263443

[B187] Schilder C. M. Seynaeve C. Beex L. V. Boogerd W. Linn S. C. Gundy C. M. (2010). Effects of Tamoxifen and Exemestane on Cognitive Functioning of Postmenopausal Patients with Breast Cancer: Results from the Neuropsychological Side Study of the Tamoxifen and Exemestane Adjuvant Multinational Trial. Jco 28 (8), 1294–1300. 10.1200/JCO.2008.21.3553 20142601

[B188] Schilder C. M. T. Seynaeve C. Linn S. C. Boogerd W. Beex L. V. A. M. Gundy C. M. (2012). Self-reported Cognitive Functioning in Postmenopausal Breast Cancer Patients before and during Endocrine Treatment: Findings from the Neuropsychological TEAM Side-Study. Psycho-Oncology 21 (5), 479–487. 10.1002/pon.1928 21351188

[B189] Schmidt J. E. Beckjord E. Bovbjerg D. H. Low C. A. Posluszny D. M. Lowery A. E. (2016). Prevalence of Perceived Cognitive Dysfunction in Survivors of a Wide Range of Cancers: Results from the 2010 LIVESTRONG Survey. J. Cancer Surviv 10 (2), 302–311. 10.1007/s11764-015-0476-5 26238504 PMC5772767

[B190] Schmitz K. H. Courneya K. S. Matthews C. Demark-Wahnefried W. Galvão D. A. Pinto B. M. (2010). American College of Sports Medicine Roundtable on Exercise Guidelines for Cancer Survivors. Med. Sci. Sports Exerc. 42 (7), 1409–1426. 10.1249/MSS.0b013e3181e0c112 20559064

[B191] Seigers R. Fardell J. E. (2011). Neurobiological Basis of Chemotherapy-Induced Cognitive Impairment: a Review of Rodent Research. Neurosci. Biobehavioral Rev. 35 (3), 729–741. 10.1016/j.neubiorev.2010.09.006 20869395

[B192] Seigers R. Schagen S. B. Van Tellingen O. Dietrich J. (2013). Chemotherapy-related Cognitive Dysfunction: Current Animal Studies and Future Directions. Brain Imaging Behav. 7 (4), 453–459. 10.1007/s11682-013-9250-3 23949877

[B193] Seliktar N. Polek C. Brooks A. Hardie T. (2015). Cognition in Breast Cancer Survivors: Hormones versus Depression. Psycho-Oncology 24 (4), 402–407. 10.1002/pon.3602 25044780

[B194] Sharafeldin N. Bosworth A. Patel S. K. Chen Y. Morse E. Mather M. (2018). Cognitive Functioning after Hematopoietic Cell Transplantation for Hematologic Malignancy: Results from a Prospective Longitudinal Study. Jco 36 (5), 463–475. 10.1200/JCO.2017.74.2270 29252122

[B195] Sharon G. Cruz N. J. Kang D.-W. Gandal M. J. Wang B. Kim Y.-M. (2019). Human Gut Microbiota from Autism Spectrum Disorder Promote Behavioral Symptoms in Mice. Cell 177 (6), 1600–1618. 10.1016/j.cell.2019.05.004 31150625 PMC6993574

[B196] Sheinerman K. S. Tsivinsky V. G. Crawford F. Mullan M. J. Abdullah L. Umansky S. R. (2012). Plasma microRNA Biomarkers for Detection of Mild Cognitive Impairment. Aging 4 (9), 590–605. 10.18632/aging.100486 23001356 PMC3492224

[B197] Shibayama O. Yoshiuchi K. Inagaki M. Matsuoka Y. Yoshikawa E. Sugawara Y. (2014). Association between Adjuvant Regional Radiotherapy and Cognitive Function in Breast Cancer Patients Treated with Conservation Therapy. Cancer Med. 3 (3), 702–709. 10.1002/cam4.174 24756915 PMC4101762

[B198] Shibayama O. Yoshiuchi K. Inagaki M. Matsuoka Y. Yoshikawa E. Sugawara Y. (2019). Long-term Influence of Adjuvant Breast Radiotherapy on Cognitive Function in Breast Cancer Patients Treated with Conservation Therapy. Int. J. Clin. Oncol. 24 (1), 68–77. 10.1007/s10147-018-1330-3 30168090

[B199] Silva Y. P. Bernardi A. Frozza R. L. (2020). The Role of Short-Chain Fatty Acids from Gut Microbiota in Gut-Brain Communication. Front. Endocrinol. 11, 25. 10.3389/fendo.2020.00025 PMC7005631 32082260

[B200] Simó M. Root J. C. Vaquero L. Ripollés P. Jové J. Ahles T. (2015). Cognitive and Brain Structural Changes in a Lung Cancer Population. J. Thorac. Oncol. 10 (1), 38–45. 10.1097/JTO.0000000000000345 25325778 PMC5657249

[B201] Singh P. Yan J. Hull R. Read S. O'Sullivan J. Henderson R. D. (2011). Levels of Phosphorylated Axonal Neurofilament Subunit H (pNfH) Are Increased in Acute Ischemic Stroke. J. Neurol. Sci. 304 (1-2), 117–121. 10.1016/j.jns.2011.01.025 21349546

[B202] Skaali T. Fosså S. D. Andersson S. Cvancarova M. Langberg C. W. Lehne G. (2011). A Prospective Study of Neuropsychological Functioning in Testicular Cancer Patients. Ann. Oncol. 22 (5), 1062–1070. 10.1093/annonc/mdq553 21048038

[B203] Small B. J. Rawson K. S. Walsh E. Jim H. S. L. Hughes T. F. Iser L. (2011). Catechol-O-methyltransferase Genotype Modulates Cancer Treatment-Related Cognitive Deficits in Breast Cancer Survivors. Cancer 117 (7), 1369–1376. 10.1002/cncr.25685 21425136

[B204] Stouten-Kemperman M. M. de Ruiter M. B. Caan M. W. A. Boogerd W. Kerst M. J. Reneman L. (2015). Lower Cognitive Performance and white Matter Changes in Testicular Cancer Survivors 10 Years after Chemotherapy. Hum. Brain Mapp. 36 (11), 4638–4647. 10.1002/hbm.22942 26304182 PMC6869574

[B205] Tager F. A. McKinley P. S. Schnabel F. R. El-Tamer M. Cheung Y. K. K. Fang Y. (2010). The Cognitive Effects of Chemotherapy in post-menopausal Breast Cancer Patients: a Controlled Longitudinal Study. Breast Cancer Res. Treat. 123 (1), 25–34. 10.1007/s10549-009-0606-8 19894112

[B206] Takeuchi O. Akira S. (2010). Pattern Recognition Receptors and Inflammation. Cell 140 (6), 805–820. 10.1016/j.cell.2010.01.022 20303872

[B207] Thiery-Vuillemin A. Poulsen M. H. Lagneau E. Ploussard G. Birtle A. Dourthe L.-M. (2020). Impact of Abiraterone Acetate Plus Prednisone or Enzalutamide on Patient-Reported Outcomes in Patients with Metastatic Castration-Resistant Prostate Cancer: Final 12-mo Analysis from the Observational AQUARiUS Study. Eur. Urol. 77 (3), 380–387. 10.1016/j.eururo.2019.09.019 31594705

[B208] van der Flier W. M. Schoonenboom S. N. M. Pijnenburg Y. A. L. Fox N. C. Scheltens P. (2006). The Effect of APOE Genotype on Clinical Phenotype in Alzheimer Disease. Neurology 67 (3), 526–527. 10.1212/01.wnl.0000228222.17111.2a 16894123

[B209] van der Willik K. D. Koppelmans V. Hauptmann M. Compter A. Ikram M. A. Schagen S. B. (2018). Inflammation Markers and Cognitive Performance in Breast Cancer Survivors 20 Years after Completion of Chemotherapy: a Cohort Study. Breast Cancer Res. 20 (1), 135. 10.1186/s13058-018-1062-3 30442190 PMC6238315

[B210] Van Dyk K. Crespi C. M. Bower J. E. Castellon S. A. Petersen L. Ganz P. A. (2019). The Cognitive Effects of Endocrine Therapy in Survivors of Breast Cancer: A Prospective Longitudinal Study up to 6 Years after Treatment. Cancer 125 (5), 681–689. 10.1002/cncr.31858 30485399 PMC6378114

[B211] Vardy J. Dhillon H. M. Pond G. R. Rourke S. B. Xu W. Dodd A. (2014). Cognitive Function and Fatigue after Diagnosis of Colorectal Cancer. Ann. Oncol. 25 (12), 2404–2412. 10.1093/annonc/mdu448 25214544 PMC4239806

[B212] Vardy J. L. Bray V. J. Dhillon H. M. (2017). Cancer-induced Cognitive Impairment: Practical Solutions to Reduce and Manage the challenge. Future Oncol. 13 (9), 767–771. 10.2217/fon-2017-0027 28266247

[B213] Vardy J. L. Dhillon H. M. Pond G. R. Rourke S. B. Bekele T. Renton C. (2015). Cognitive Function in Patients with Colorectal Cancer Who Do and Do Not Receive Chemotherapy: A Prospective, Longitudinal, Controlled Study. Jco 33 (34), 4085–4092. 10.1200/JCO.2015.63.0905 PMC5683012 26527785

[B214] Vardy J. Tannock I. (2007). Cognitive Function after Chemotherapy in Adults with Solid Tumours. Crit. Rev. Oncology/Hematology 63 (3), 183–202. 10.1016/j.critrevonc.2007.06.001 17678745

[B215] Von Ah D. Carpenter J. S. Saykin A. Monahan P. Wu J. Yu M. (2012). Advanced Cognitive Training for Breast Cancer Survivors: a Randomized Controlled Trial. Breast Cancer Res. Treat. 135 (3), 799–809. 10.1007/s10549-012-2210-6 22918524 PMC3677054

[B216] Von Ah D. Crouch A. (2020). Cognitive Rehabilitation for Cognitive Dysfunction after Cancer and Cancer Treatment: Implications for Nursing Practice. Semin. Oncol. Nurs. 36 (1), 150977. 10.1016/j.soncn.2019.150977 31959511 PMC7853869

[B217] Von Ah D. Russell K. M. Storniolo A. M. Carpenter J. S. (2009). Cognitive Dysfunction and its Relationship to Quality of Life in Breast Cancer Survivors. Oncol. Nurs. Forum 36 (3), 326–336. 10.1188/09.ONF.326-334 19596650

[B218] Wagner L. I. Gray R. J. Sparano J. A. Whelan T. J. Garcia S. F. Yanez B. (2020). Patient-Reported Cognitive Impairment Among Women with Early Breast Cancer Randomly Assigned to Endocrine Therapy Alone versus Chemoendocrine Therapy: Results from TAILORx. Jco 38 (17), 1875–1886. 10.1200/JCO.19.01866 PMC7280048 32271671

[B219] Wagner L. Sweet J. Butt Z. Lai J. S. Cella D. (2009). Measuring Patient Self-Reported Cognitive Function: Development of the Functional Assessment of Cancer Therapy–Cognitive Function Instrument. J. Support. Oncol. 7 (6), W32–W39.

[B220] Ward A. Crean S. Mercaldi C. J. Collins J. M. Boyd D. Cook M. N. (2012). Prevalence of apolipoprotein E4 genotype and homozygotes (APOE e4/4) among patients diagnosed with Alzheimer's disease: a systematic review and meta-analysis. Neuroepidemiology 38 (1), 1–17. 10.1159/000334607 22179327

[B221] Weber A. Wasiliew P. Kracht M. (2010). Interleukin-1 (IL-1) Pathway. Sci. Signal. 3 (105), cm1. 10.1126/scisignal.3105cm1 20086235

[B222] Wefel J. S. Lenzi R. Theriault R. L. Davis R. N. Meyers C. A. (2004). The Cognitive Sequelae of Standard-Dose Adjuvant Chemotherapy in Women with Breast Carcinoma. Cancer 100 (11), 2292–2299. 10.1002/cncr.20272 15160331

[B223] Wefel J. S. Saleeba A. K. Buzdar A. U. Meyers C. A. (2010). Acute and Late Onset Cognitive Dysfunction Associated with Chemotherapy in Women with Breast Cancer. Cancer 116 (14), 3348–3356. 10.1002/cncr.25098 20564075

[B224] Wefel J. S. Vardy J. Ahles T. Schagen S. B. (2011). International Cognition and Cancer Task Force Recommendations to Harmonise Studies of Cognitive Function in Patients with Cancer. Lancet Oncol. 12 (7), 703–708. 10.1016/S1470-2045(10)70294-1 21354373

[B225] Wefel J. S. Vidrine D. J. Marani S. K. Swartz R. J. Veramonti T. L. Meyers C. A. (2014). A Prospective Study of Cognitive Function in Men with Non-seminomatous Germ Cell Tumors. Psycho-Oncology 23 (6), 626–633. 10.1002/pon.3453 24339329 PMC4066616

[B226] Wen J. Maxwell R. R. Wolf A. J. Spira M. Gulinello M. E. Cole P. D. (2018). Methotrexate Causes Persistent Deficits in Memory and Executive Function in a Juvenile Animal Model. Neuropharmacology 139, 76–84. 10.1016/j.neuropharm.2018.07.007 29990472 PMC6089371

[B227] Whitford H. S. Kalinowski P. Kalinowski P. Schembri A. Grimison P. Stockler M. (2020). The Impact of Chemotherapy on Cognitive Function: a Multicentre Prospective Cohort Study in Testicular Cancer. Support Care Cancer 28 (7), 3081–3091. 10.1007/s00520-019-05095-3 31642990

[B228] Wiechno P. J. Sadowska M. Kalinowski T. Michalski W. Demkow T. (2013). Does Pharmacological Castration as Adjuvant Therapy for Prostate Cancer after Radiotherapy Affect Anxiety and Depression Levels, Cognitive Functions and Quality of Life? Psycho-Oncology 22 (2), 346–351. 10.1002/pon.2095 22081540

[B229] Williams A. M. Shah R. Shayne M. Huston A. J. Krebs M. Murray N. (2018). Associations between Inflammatory Markers and Cognitive Function in Breast Cancer Patients Receiving Chemotherapy. J. Neuroimmunology 314, 17–23. 10.1016/j.jneuroim.2017.10.005 29128118 PMC5768199

[B230] Winocur G. Wojtowicz J. M. Huang J. Tannock I. F. (2014). Physical Exercise Prevents Suppression of Hippocampal Neurogenesis and Reduces Cognitive Impairment in Chemotherapy-Treated Rats. Psychopharmacology 231 (11), 2311–2320. 10.1007/s00213-013-3394-0 24343419

[B231] Wouters H. Baars J. W. Schagen S. B. (2016). Neurocognitive Function of Lymphoma Patients after Treatment with Chemotherapy. Acta Oncologica 55 (9-10), 1121–1125. 10.1080/0284186X.2016.1189092 27333078

[B232] Xie B. Liu Z. Jiang L. Liu W. Song M. Zhang Q. (2016). Increased Serum miR-206 Level Predicts Conversion from Amnestic Mild Cognitive Impairment to Alzheimer's Disease: A 5-Year Follow-Up Study. Jad 55 (2), 509–520. 10.3233/JAD-160468 27662297

[B233] Xie B. Zhou H. Zhang R. Song M. Yu L. Wang L. (2015). Serum miR-206 and miR-132 as Potential Circulating Biomarkers for Mild Cognitive Impairment. Jad 45 (3), 721–731. 10.3233/JAD-142847 25589731

[B234] Yamamoto S. Masutani E. Arao H. (2020). Self-reported Cognitive Decline in Japanese Patients with Breast Cancer Treated with Endocrine Therapy. Breast Cancer 27 (4), 670–682. 10.1007/s12282-020-01062-7 32114664

[B235] Yao C. Rich J. B. Tannock I. F. Seruga B. Tirona K. Bernstein L. J. (2016). Pretreatment Differences in Intraindividual Variability in Reaction Time between Women Diagnosed with Breast Cancer and Healthy Controls. J. Int. Neuropsychol. Soc. 22 (5), 530–539. 10.1017/S1355617716000126 26960672

[B236] Zahodne L. Manly J. Narkhede A. Griffith E. DeCarli C. Schupf N. (2015). Structural MRI Predictors of Late-Life Cognition Differ across African Americans, Hispanics, and Whites. Car 12 (7), 632–639. 10.2174/1567205012666150530203214 PMC4872300 26027808

[B237] Zhang L. Wang Y. Xiayu X. Shi C. Chen W. Song N. (2017). Altered Gut Microbiota in a Mouse Model of Alzheimer's Disease. Jad 60 (4), 1241–1257. 10.3233/JAD-170020 29036812

[B238] Zhang X. Yuan X. Shi H. Wu L. Qian H. Xu W. (2015). Exosomes in Cancer: Small Particle, Big Player. J. Hematol. Oncol. 8, 83. 10.1186/s13045-015-0181-x 26156517 PMC4496882

